# Heterogeneity in The Mechanical Properties of Integrins Determines Mechanotransduction Dynamics in Bone Osteoblasts

**DOI:** 10.1038/s41598-019-47958-z

**Published:** 2019-09-11

**Authors:** Aban Shuaib, Daniyal Motan, Pinaki Bhattacharya, Alex McNabb, Timothy M. Skerry, Damien Lacroix

**Affiliations:** 1 Department of Oncology and Metabolism, University of Sheffield, Sheffield, UK; 2 Insigneo Institute for in silico Medicine, University of Sheffield, Sheffield, UK; 3 Department of Mechanical Engineering, University of Sheffield, Sheffield, UK

**Keywords:** Emergence, Computational models

## Abstract

Bone cells are exposed to dynamic mechanical stimulation that is transduced into cellular responses by mechanotransduction mechanisms. The extracellular matrix (ECM) provides a physical link between loading and bone cells, where mechanoreceptors, such as integrins, initiate mechanosensation. Though this relationship is well studied, the dynamic interplay between mechanosensation, mechanotransduction and cellular responses is unclear. A hybrid-multiscale model combining molecular, cellular and tissue interactions was developed to examine links between integrins’ mechanosensation and effects on mechanotransduction, ECM modulation and cell-ECM interaction. The model shows that altering integrin mechanosensitivity threshold (MT) increases mechanotransduction durations from hours to beyond 4 days, where bone formation starts. This is relevant to bone, where it is known that a brief stimulating period provides persistent influences for over 24 hours. Furthermore, the model forecasts that integrin heterogeneity, with respect to MT, would be able to induce sustained increase in pERK baseline > 15% beyond 4 days. This is analogous to the emergence of molecular mechanical memory signalling dynamics. Therefore, the model can provide a greater understanding of mechanical adaptation to differential mechanical responses at different times. Given reduction of bone sensitivity to mechanical stimulation with age, these findings may lead towards useful therapeutic targets for upregulation of bone mass.

## Introduction

Mechanical stimulation plays an important role in shaping bone’s architecture, mechanical properties and resistance failure^1–3^. Bone cells such as osteoblasts (OBs) and osteocytes (OCs) are involved in bone maintenance in health and disease. Translation of mechanical cues to cellular responses is mediated through mechanosensation and mechanotransduction. Both cell types can detect mechanical stimulation (forces applied to the whole bone, inducing tissue strain and fluid flow through the matrix) via anchorage proteins (mechanoreceptors) that provide a direct physical link between the cells and bone tissue^4–6^. Integrins are the main mechanoreceptors expressed by OBs and OCs and they are indispensable for bone functionality^7^. Integrins were shown to transduce mechanical forces to cellular response via biochemical cascades and cytoskeletal modification^8^. Their mutation, depletion or inhibition antagonises osteogenesis *in vitro*. *In vivo*, mice lacking the β1 integrin gene have a phenotype that includes skeletal defects, while stimulating α5β1 integrins in mice induce bone repair^9–13^. In addition, OB-mediated extracellular matrix (ECM) mineralisation is driven by integrins^14^. Furthermore, during osteogenesis and OB differentiation, gene expression of the α5 and β1 integrin subunits is enhanced^15,16^. Complexity of integrin-mediated mechanotransduction is multifaceted as the protiens can form 24 possible functional dimers^17^ distinct in their mechanosensitivity and elicited cellular responses^10,18–20^. Each dimer can form diverse complexes with multiple intracellular adaptor proteins^21^ to dictate the interplay between biochemical and cytoskeletal elements, and consequently determine their contribution to cellular mechano-response^22,23^. Additionally integrins expression is variable with respect to cellular location and through an OB’s lifespan^24,25^.

As mechanosensation is dependent on ECM material properties such as stiffness, the matrix can alter transfer of mechanical stimulation from tissue to cellular and subcellular levels. It has been demonstrated experimentally and computationally that ECM stiffness determines cellular response, for instance during mesenchymal stem cell differentiation to OBs^26–28^. Numerous mathematical in silico models, using continuum and microscale approaches, have been used to investigate the link between ECM stiffness and cellular response^29–33^. They illustrate the dependence of cellular responses on cytoskeletal element composed of actin fibres and myosin proteins^34^. Furthermore, they demonstrate how the cellular response alters the ECM’s mechanical stiffness. Although those models are successful in predicting cellular responses in line with experimental results, they have limitations. Many are one-dimensional models; their predictions are within narrow limits, and do not yield emergent complex behaviours such as differentiation or molecular memory. Furthermore, models designed to address mechanotransduction by coupling cytoskeletal elements with mechanotransduction biochemical pathways are sparse. A model by Sun *et al*. included mechanotransduction events leading to the recruitment of YAP-TAZ transcription factors (TFs). It demonstrated inclusion of biochemical events, further highlighting the importance of understanding the relationships between mechanosensation, cellular response and interaction with the ECM^35^. Dingal and Discher elucidated how modulation of gene expression events linked to ECM collagen fibres and myosin II, had long term effects on ECM stiffness and cell spreading^36^.

Therefore, differential modulation of integrin mechanical properties can give rise to distinctive mechanotransduction dynamics which ultimately affect the ECM stiffness. That in turn imposes new conditions which potentially influence OB differentiation decisions. This is believed to be related to cells retention of mechanical memory of previous applied tissue load (AF_T_)^37–40^. To date, a complete understanding of the interplay between active mechanosensing, mechanotransduction and modulation of the ECM material properties remain elusive. This paper investigates the following: the impact modulation of integrins mechanosensitivity, and their population heterogeneity, have on mechanotransduction dynamics; whether this impact is influenced by specific ratios between integrins with different mechaosensitive properties; and finally, what are the consequences on OB response to mechanical stimulation and modulation of tissue material properties. This study illustrates that the mechanosensing properties of integrins are important for maintaining response to AF_T_. Additionally, it has shown that mechanical molecular memory of previous mechanical signals can emerge due to the presence of heterogeneous integrins population containing 1% of individuals ultrasensitive to AF_T_.

## Results

**Figure 1:**
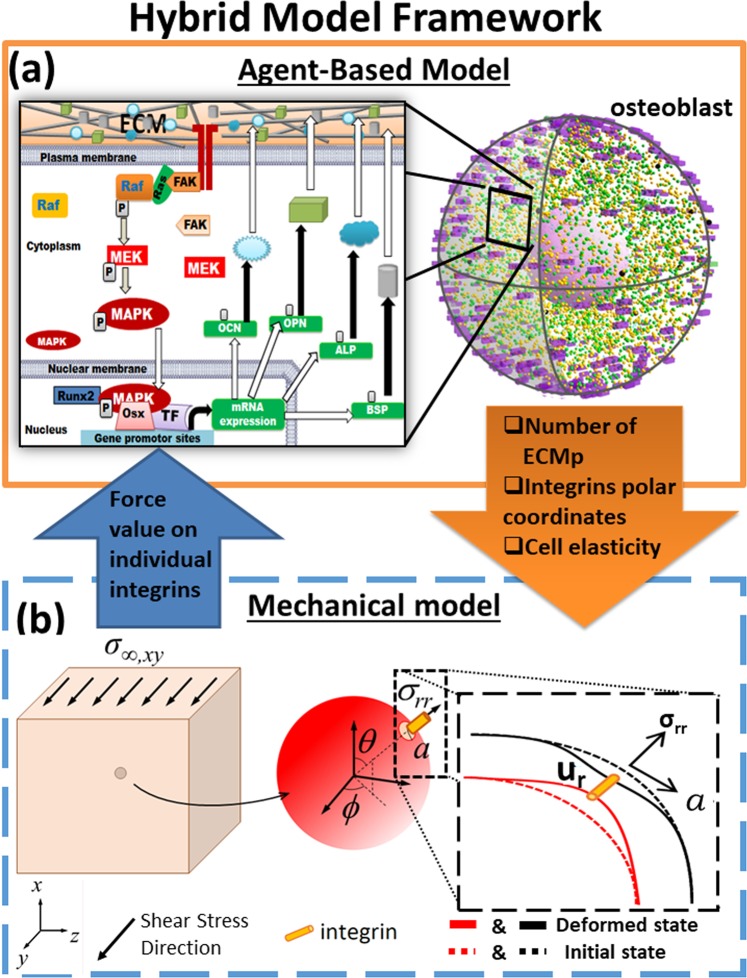
A hybrid multi-scale model of mechanotransduction combining an agent based model (ABM) and a mechanical model. (**a**) The ABM simulated molecular events downstream of integrin mechanoreceptors in a spherical osteoblast responding to mechanical stimulation imposed at the ECM. The enlarged box shows the cascade simulated by the ABM which focused on biochemical mechanotransduction. This recruited the ERK pathway and its induced osteogenic gene expression events. These involved TF Runx2 recruitment, and its ensemble to express osteogenic ECM genes. Their corresponding mRNA and proteins were produced and the latter were deposited in the surrounding ECM. (**b**) A representation of the mechanical model; at the tissue level a unidirectional and constant shear stress was applied and was propagated through the ECM. The mechanical force was transferred to the interface between the cell and the ECM, resulting in cell and plasma membrane compression. Integrins resided at the interface, and the opposing stretch forces at the ECM and the plasma membrane were triggers for integrin activation. The values of the forces were computed and quantified by the mechanical model. The enlarged box illustrates the required parameters to calculate the forces generated on individual integrin-agents. The two models communicated every iteration which was equivalent to 1 s. The arrows between the two models represent these communications and the input/output parameters. ECM, ECMp FAK, Ras, Raf, MEK, MAPK, Runx2, Osx, TF, ALP, OCN, OPN, BSP, U_r_, r, θ and ϕ stand for: extracellular matrix, extracellular matrix protein, focal adhesion kinase, Rat sarcoma, Rapidly Accelerated Fibrosarcoma protein, Mitogen-activated protein kinase kinase, Mitogen-activated protein kinase, Runt-related transcription factor 2, Ostrix, transcription factor, alkaline phosphatase, osteocalcin, osteopontin, bone sialoprotein, deformation of cell and ECM surfaces, and spherical polar coordinates locations respectively.

Modulation of integrins’ mechanosensitivity to AF_T_ and its impact on mechanotransduction, and consequently, synthesis of osteogenic proteins was investigated via a hybrid model which coupled a mechanical model and an Agent-Based Model (ABM), hence referred to as Mech-ABM (Fig. 1).

The key principles of Mech-ABM are outlined here: The applied tissue load (AF_T_), which was modelled as a unidirectional constant shear stress, propagates through the ECM to reach the cell’s plasma membrane through the anchorage proteins and mechanoreceptors at the interface with the ECM. Integrins are the primary anchorage proteins and mechanoreceptors at the plasma membrane. AF_T_ stretches the integrins, modelled as linear springs, and therefore change their confirmation leading to their priming for full activation. Mechanical excitation drives OB differentiation to OCy and the expression of osteogenic markers such as alkaline phosphatase (ALP), osteopontin (OCP), osteocalcin (OCN) and Bone sialoprotein (BSP), which are also extracellular matrix proteins (ECMp). Each ECMp modulates the material properties of the surrounding ECM by increasing its stiffness. Subsequently transfer of AF_T_ changes due to reduced ECM stretching and therefore mechanosensation via integrins is altered. Integrin activation initiates intracellular mechanotransduction and provides the link between the mechanical and ABM models.

**Figure 2:**
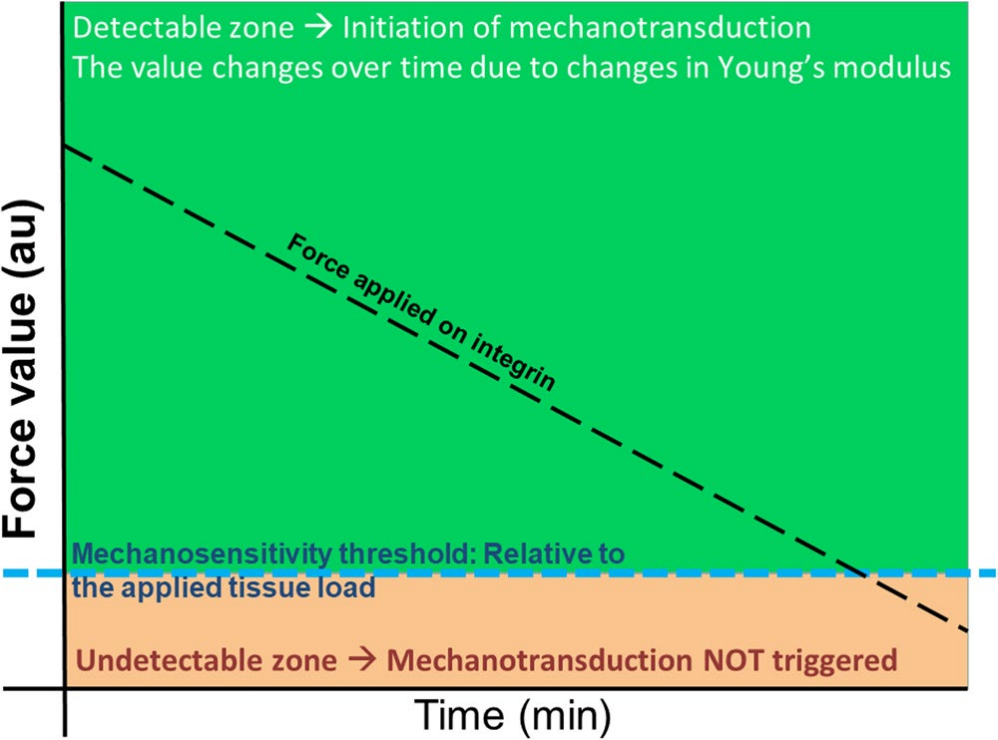
The principle of integrin mechanosensitivity threshold (MT). At the tissue level, the bone is exposed to mechanical loading, which is transferred through the ECM to cells via the integrin mechanoreceptors. Integrins were shown to sense forces at values which are 2–10 pN and are proposed to have an activation threshold for sensing the ECM stiffness^41^. The MT in the Mech-ABM was set to a value proportional to applied AF_T_ (blue dotted line). MT was a memory variable for each integrin-agent in the ABM. The mechanical model calculated the numerical value of force applied on individual integrin-agents. Any force value that was equal to or above MT (the green area) triggered integrin activation and initiated mechanotransduction events downstream integrins which satisfied this rule. Force values that were below MT (the orange area) were undetectable by integrins which were, therefore, not activated; consequently, no mechanotransduction initiation occurred downstream of these insensitive integrins. The black dotted line demonstrates the reduction of the force magnitude applied on integrins due to increased stiffness of the ECM.

The ABM simulated mechanotransduction events downstream of integrins and focal adhesion kinase (FAK) protein within a spherical OB in a 3D ECM (osteoid, Fig. 1(a)). The main assumptions within the ABM are summarised below:

- The intracellular molecules were homogenously distributed within their appropriate compartments (cytoplasmic and nuclear compartments) in a well-mixed solution.
- As in the physiological scenario, mechanotransduction is driven by molecule-molecule interaction, which are affected by diffusion; the molecules availability for chemical interaction and modification; and cyclic transitioning between active and dormant states.
- The molecular interactions were modelled stochastically as intracellular biochemical events, focusing on the extracellular signal-regulated kinase (ERK) cascade.
- The ERK cascade is heavily regulated by multiple mechanisms, which are not within the scope of the model and therefore were treated via black-box modelling approach.
- Every molecule was modelled as an autonomous agent which diffuses by Brownian motion. Integrins were modelled as static agents homogenously distributed within the plasma membrane.
- Exposure to AF_T_ did not lead to further integrin recruitment, and neither altered their mechanical properties. Their mechanosensitivities were modified state variables that specified their threshold to AF_T_ (Fig. 2).
- The ERK pathway is the principal pathway linking mechanotransduction and osteogenic differentiation.
- Mechanotransduction activation dynamics were represented as magnitudes of activated integrins, ERK and level of the osteogenic ECMps ALP, OCP, OCN and BSP. The mechanical model simulated and computed the biomechanics at the tissue level, where the tissue was exposed to a constant unidirectional shear stress. The model quantified the forces exerted at specific areas on the plasma membrane, where integrins resided (Fig. 1(b)).

### A ten-fold change in integrin mechanosensitivity threshold (MT) determines the duration of mechanotransduction

**Figure 3:**
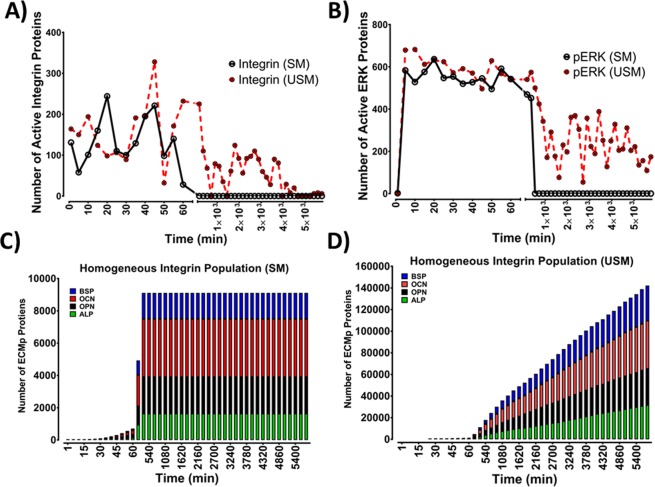
Alteration of the integrin mechanosensitivity threshold impacts mechanotransduction dynamics and maintenance. Representation of the mechanotransduction behaviour of Sensitive (SM) and Ultrasensitive (USM) Mech-ABMs within the initial 60 min and beyond 8 h of mechanical stimulation. (**A**) Integrin activation dynamics were not statistically different in SM and USM during the initial 60 min of activation. Integrin population in the SM were activated by the mechanical stimulus in the initial phase (0–60 min), this activation behaviour was followed by gradual reduction leading to complete inactivity within the initial 180 ± 20 min. Conversely, in USM, integrins detection and activation by mechanical stimulation was maintained beyond 96 h (4 days). (**B**) Mechanotransduction events were examined via monitoring phosphorylated ERK (pERK). In SM, mechanotransduction was maintained until 107 min. Contrariwise, in USM, pERK generation was maintained past 96 h (4 days), although levels were approximately 50% less compared to the initial phase of activation (0–60 min). (**C**) Stacked bars demonstrating accumulation of the four ECMps (OPN, OCN, ALP and OCN) over 4 Days of mechanical stimulation. The initial activation phase showed no statistical difference between the two models. However, after 150 min levels of ECMp plateau in SM, while ECMp levels continue to increase in the USM. Mechanical stimulation at the tissue level was a constant unidirectional shear stress. OPN, OCN, ALP and BSP abbreviate osteopontin, osteocalcin, alkaline phosphatase and bone sialoprotein respectively. Data shown from one simulation (n = 1). Sensitive model refers to Mech-ABM where integrin mechanosensitivity threshold was 10% of applied force at the tissue level (AF_T_), while the Ultrasensitive was 1% of AF_T_.

Modulation of integrins mechanosensitivity to applied force and its impact on mechanotransduction and synthesis of osteogenic proteins were investigated via four models (Table S1). Different Integrin dimers exhibit diverse mechanosensitivity characteristics, these are mostly reported in the literature via qualitative measures and there is no direct quantification of AF_T_. Nonetheless, Elosegui-Artola *et al*. have shown that integrin mechanosensation is related to a threshold relative to AF_T_^41^. MT was defined as a numerical value of mechanical load that an integrin is exposed to: If AF_T_’s value was equal or above MT, it leads to integrins activation (Fig. 2). Initially modulation of MT was examined using two models where integrin populations were homogeneous with respect to MT. In the first model MT was set to 10% of AF_T_ (sensitive model (SM)); the second MT was set to 1% of AF_T_ (ultrasensitive model (USM)). The two models had no statistically significant difference during their initial activation phases (Fig. 3), with both models displaying similar distribution of active integrins in the initial 70 min (Fig. S1). However, after 2 h, the two models diverged; with no active integrins remaining in the SM, while in the USM, integrin activation persisted even beyond 96 h (4 days) of stimulation (Fig. 3(A)). Nonetheless, in the USM, active integrin levels were significantly reduced in comparison to the initial 70 min of activation (Fig. S2). Full integrin activation initiates mechanotransduction by activating the FAK arm which stimulates the ERK pathway, which is considered as the main osteogenic signalling pathway. Therefore, the impact of modulating integrins’ mechanosensitivity on mechanotransduction events was examined via monitoring pERK activation dynamics (Fig. 3(B)). In the initial 60 min, pERK levels were not statistically significant (Fig. S3). Nonetheless, with SM pERK levels started to decline at 93 ± 10 min, and reduced to 0 at 360 ± 7 min. Conversely, using the USM ERK activation was sustained and continued past 4 days, nonetheless these pERK levels were significantly lower compared to observations in the initial 60 min (Fig. S4).

ERK initiates osteogenic gene expression events leading to expression and secretion of the osteogenic markers, these are also ECMp. Thus, the effect of altering integrins mechanosensitivity on mechanotransduction was further assessed via monitoring levels of deposited osteogenic ECMps. With the SM, synthesis of the corresponding ECMps was significantly reduced and eventually plateaued (Fig. 3(C)). However, when the USM was examined, ECMp synthesis and deposition continued past 4 days (Fig. 3(D)). These results demonstrated that modulation of integrin sensitivity to mechanical load, impacts the duration of mechanotransduction and protein synthesis. Moreover, those results illustrated that the relation between integrin mechanosensitivity and mechanotransduction is monotonic.

### A fraction of integrin population is required for mechanotransduction maintenance and extracellular matrix proteins synthesis and deposition

**Figure 4:**
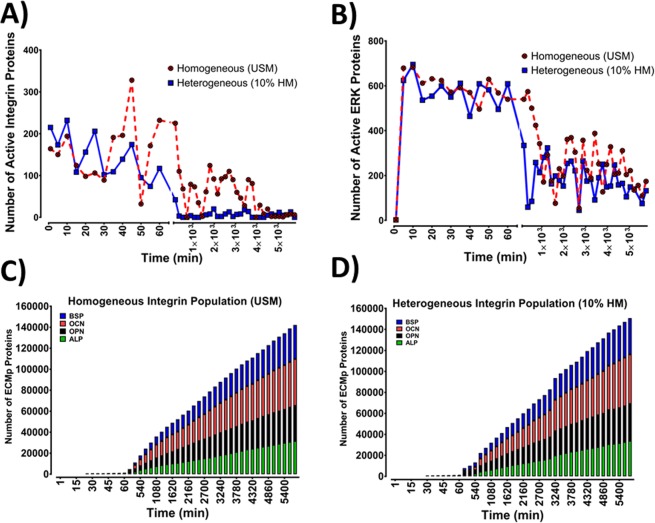
Comparisons between the ultrasensitive model (USM) and the heterogeneous model (10%-HM) of integrin mechanosensitivity, revealed that mechanotransduction maintenance depends on a fraction of total integrin population. (**A**) Illustration of the integrins activation behaviour of USM and 10%-HM within the initial 60 min and beyond 8 h of mechanical stimulation. At 0–60 min, the two models demonstrated similar activation behaviour and statistical analysis illustrated no significant difference between the two models. Response to mechanical stimulus and integrin activation was persistent past 4 days, nonetheless, the level of active integrins mediating mechanotransduction events were significantly lower in 10%-HM. (**B**) Mechanotransduction presented as pERK levels. At the initial phase (0–60 min), both models exhibited similar pERK activation dynamics where pERK maximal response was achieved during the first 7 ± 2 min after mechanical stimulation was applied, and pERK levels were sustained during the initial 60 min. Statistical analysis indicated no significant difference between the two models. After 16 hours, pERK levels were significantly reduced, yet mechanotransduction and pERK activation were maintained beyond 96 h (4 days). pERK levels were statistically lower in the 10%-HM in comparison to the homogeneous sensitive model. (**C**,**D**) Stacked bars illustrating the levels of accumulated ECMp overtime, it displays the four ECMps (OPN, OCN, ALP and BSP) at hourly intervals. There was no statistically significant difference  between the two models for the four ECMps. Mechanical stimulation at the tissue level was a constant unidirectional shear stress. Data shown from one simulation (n = 1). Ultrasensitive model refers to Mech-ABM where integrin mechanosensitivity threshold was 1% of AF_T_, while the 10%-HM is a heterogenous model where the ratio between ultrasensitive and sensitive integrins was 1:10.

The impact of mechanosensitivity heterogeneity, within an integrin population, on mechanotransduction and ECMp deposition were investigated using two models, where integrin populations were heterogeneous (HM). This was achieved by combining ultrasensitive and sensitive integrins within one population. In one model, the integrin population ratio was 1:10 whereby for 1 ultrasensitive integrin there were 10 sensitive integrins (10%-HM); in the other, the ultrasensitive-to-sensitive heterogeneity was changed to 1:100 ratios respectively (1%-HM). The ratios were chosen based on Elosegui-Artola *et al*. who demonstrated that a fivefold ratio between two integrin populations (αvβ6 and α5β1) impacted cellular mechanosensation^41^. Firstly, the mechanotransduction dynamics were compared between the homogeneous USM and the 10%-HM. Two mechanotransduction phases emerged in the homogeneous USM. An initial phase (0–93 ± 10 min) characterised by activation of 49.8 ± 1.0% of integrins at *E*_*max*_, achieving pERK’s *E*_*max*_ and a rate of ECMp production of 13 ECMp/min (Fig. 4(A)). The second phase was typified by maintaining integrin activation, pERK formation and ECMp productions; but compared to the initial phase; there was a significant reduction in activated integrins and pERK levels, and an increased rate of ECMp synthesis (33 ECMp/min). The 10%-HM also generated biphasic activation dynamics. The initial phase was not significantly different from USM (Figs S1 and S3). Activated integrin levels after mechanical stimulation were similar for both models as observed via the distribution of active integrins during the initial 60 min (Fig. S1). However, by 16 h, the two models exhibited different activation behaviour. While the USM sustained high levels of active integrins (Figs 4(A) and S1b)), 10%-HM showed a significant drop in active integrin levels (Fig. S1b)). Active integrin levels post 16 h were significantly lower than during the initial ~65 min in 10%-HM (Figs 4(A) and S2).

Intriguingly however, when mechanotransduction was examined via assaying pERK activation dynamics, the difference between the two models had less impact than what we observed with integrin levels (Figs 4(B) and S3). In both models pERK levels were significantly lower in the second activation phase compared to the initial phase (Fig. S4). With respect to the rate of ECMp deposition, both models displayed no statistically significant difference in total ECMp number at day 4. Additionally, the rate of ECMp synthesis was not significantly different between the two models when the rate of ECMp synthesis was assayed across three activation stages (Table S1).

From these data we infer that a fraction of the integrin population with a low mechanosensitivity threshold (i.e. ultrasensitive integrins), can propagate and maintain mechanotransduction events, and can elicit maximal intracellular events.

### Emergence of mechanical memory is associated with heterogeneity of mechanosensitivity within the integrin population

**Figure 5:**
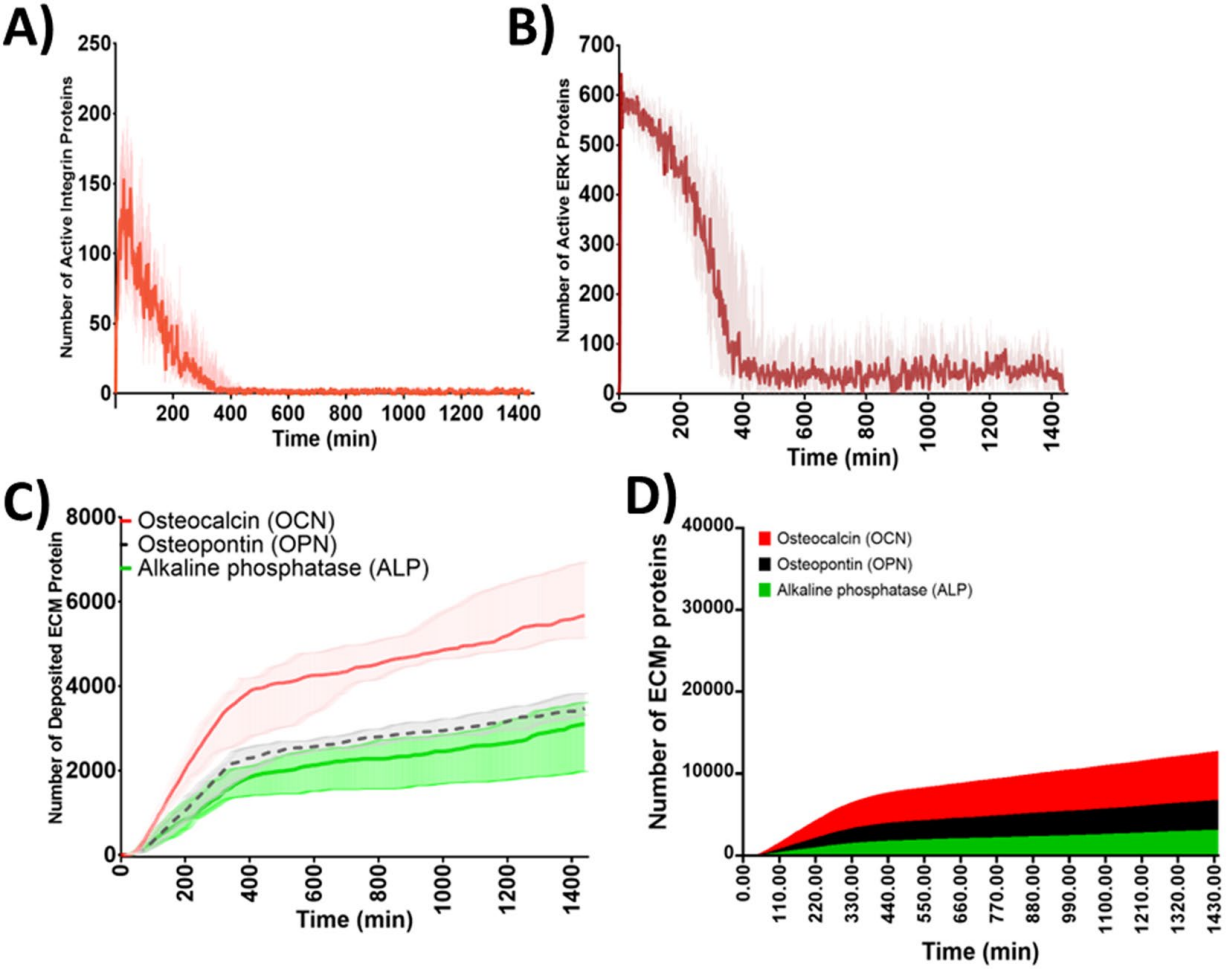
Mechanosensitivity heterogeneity in integrin population, contributes to the emergence of molecular mechanical memory of previous mechanical stimulation. The overall activation dynamics exhibited by the 1% HM Mech-ABM, during constant unidirectional shear stress mechanical stimulation. (**A**) A representation of active integrin levels over 24 h. Integrin activation was triphasic characterised by an accelerated activation at the initial phase (0–60 min), followed by deactivation phase and succeeded by a sustained activation phase which was maintained beyond 24 h. (**B**) Activated ERK (pERK) also demonstrated a triphasic activation behaviour, where the initial phase was characterised by ultrasensitive response to reach *E*_*max*_ within 6.69 ± 3 min. The maximal pERK levels were briefly maintained (100 ± 11 min) followed by gradual reduction. The third phase was characterised by the maintenance of pERK levels and the establishment of a new pERK baseline that lasted >96 h (4 days). This baseline was 80 ± 20 fold increase of pERK levels at t_0_ and 3 ± 0.8% of its *E*_*max*_. Although the 1% ultrasensitive integrin population was unable to maintain high level of pERK, however, it was capable of maintaining an adequate level of pERK beyond 24 h (and 4 days). (**C**) Graph representing the formation of and deposition of three ECMps over time from 16 simulations and represented as median ± IQR. (**D**) Stacked bars illustrating the accumulation of the four ECMps (OPN, OCN, ALP and BSP) at hourly intervals over 24 h. The points shown are from 16 simulations (n = 16) representing median ± IQR.

Using a heterogeneous integrin population comprising 1% ultrasensitive integrins (1%-HM), mechanical memory emerged (i.e. the ability for a cell to encode intracellularly previous mechanical stimulations as a form of memory to determine future responses). Residual mechanotransduction activity persisted although the mechanical load on integrins and integrin activity were insignificant compared to initial conditions at *t*_0_. The detected integrin activation dynamics were triphasic. These constituted of a rapid yet short lived activation initial phase where *E*_*max*_ was reached at 25 ± 7 min; a slow deactivation second phase; and a stable third phase (reached at 290 ± 50 min) characterised by sustained integrin activation persisting for beyond 4 days (Fig. S5a)). During the third activation phase, the active integrin levels were significantly lower than the initial phase and 5% of *E*_*max*_ or lower (12 ± 2 molecules, Fig. 5). Mechanotransduction events were examined via levels of activated pERK, and the triphasic activation dynamics were also observed. *E*_*max*_ (669 ± 27 molecules) was reached within 7 ± 2 min during the initial activation phase. This was followed by a second slow deactivation phase persisting for 340 ± 70 min, which was ensued by a sustained pERK formation post 96 h (4 days) where pERK levels were ≥ 5% of pERK’s *E*_*max*_ (Fig. S5b). Nonetheless, during the third phase, the instances where pERK levels were below 5% of pERK’s *E*_*max*_ were 35%. This activation pattern was also reflected in the number and rate of ECMp synthesised, with a 7 ± 3 molecule/min rate of synthesis in the initial stage during the initial 60 minutes, the rate significantly increased to 23 ± 9 molecules/min, then reduced to 5 ± 2 molecules/min.

## Discussion

This study presents a novel hybrid 3D model of mechanosensation and mechanotransduction (Mech-ABM) that integrates mechanoreciprocity and links mechanosensation and mechanotransduction. It demonstrated that integrins’ mechanosensing properties were important for maintaining response to applied mechanical load. The study also highlighted that heterogeneity of integrin mechanical properties are determinants of mechanotransduction dynamics. Moreover, the Mech-ABM illustrated how Integrins’ mechanosensation in conjunction with ERK pathway contributed to the emergence of mechanical memory.

**Figure 6:**
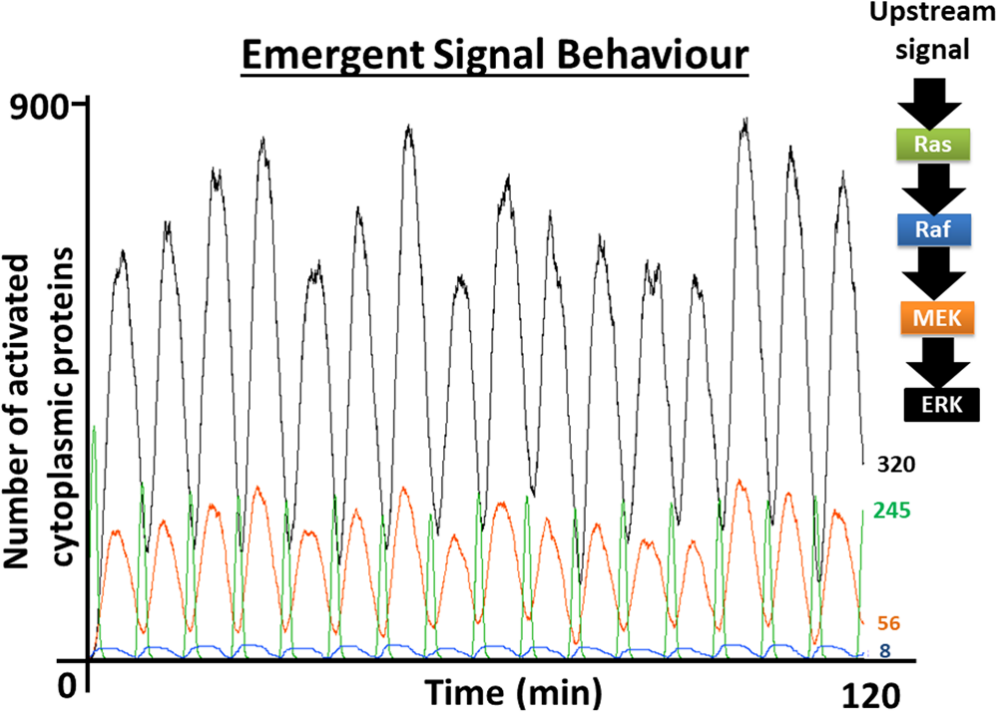
The activation dynamics of Ras and the ERK MAPK pathway as they emerged from the Mech-ABM. This demonstrated the progression of the ultrasensitive response generated in the MAPK pathway, starting from Raf and ending with ERK. The ultrasensitive response is sharpest with ERK activation and less pronounced with Raf. These activation patters are identical to those reported by Ferrell *et al*.^42^.

The Mech-ABM model simulated mechanotransduction events downstream of integrins, triggering the ERK pathway and ERK-dependent osteogenic gene expression events. These involved Runx2 activation and TFs recruitment. Some of the emergent behaviour in Mech-ABM replicated physiological behaviours previously observed in *in vitro* experimentation and in silico models (Fig. 6). These include the spare receptor theory, Raf-MEK-ERK pathway activation dynamics, and ERK oscillatory activation behaviour^42–45^. Those demonstrated Mech-ABM ability to capture physiological phenomena.

Previous in silico mechanical models and *in vitro* experimentation demonstrated that modifying focal adhesions (FAs) components altered mechanosensation behaviour and final cellular outcome^46^. Yet there is no agreement on the precise mechanical conditions and combinations within FAs to induce mesenchymal stem cells (MSCs) differentiation to particular cell type, let alone OB to OCy. Some of the proposed mechanical conditions and regimes applied are contradictory^25,47^. The Mech-ABM equally illustrated that modulation of integrin mechanosensitivity impacts mechanotransduction dynamics. Moreover, this study demonstrated that modulation of the integrin population’s mechanosensation properties influences mechanotransduction activation patterns. Therefore, it predicts that the contradictions observed experimentally can be due to differential expression of FAs with varied mechanosensitive properties at the cell membrane. This is plausible considering that Horton *et al*. demonstrated that FA, and in particular integrins, are dynamic entities under constant turnover and differentially expressed^21^. One of these is the β1 integrin subunit. Correspondingly, cell specific deletion of the β1 integrin subunit in mice bone was detrimental to osteogenesis and bone development, leading to reduced bone mass in adult mice^13^.

In agreement with Dingal *et al*., and Sun *et al*., our model suggests a nontrivial role of the soluble arm of mechanotransduction^35,36^. Previous models examining the link between mechanical stimulation and mechanosensitivity emphasised the role of FA complexes, myosin and actin filaments and their cooperation. Although it is known that adhesion complexes are comprised of several proteins which yield different mechanical properties; these are usually ignored^46,48^. This is understandable given the combinatorial complexity introduced as the number of proteins increases^49,50^. Nevertheless, the effect of modifying interactions was still observed when several mechanical models modified integrin-talin exchange rate; consequently, switching integrin binding interaction from integrin-actin to integrin-fibronectin. Hence cellular mechanosensation was modified^8,22^.

By incorporating osteogenic proteins expression events altering ECM stiffness, the Mech-ABM shed light on dynamic mechanoreciprocity between OB and the surrounding ECM. It forecasted that mechanotransduction establishes the molecular conditions allowing for subsequent mechanical-dependent cellular responses (e.g. differentiation to OCys). Sun *et al*. gene-network model also demonstrated that modulation of mechanotransduction through protein gene expression networks impact ECM stiffness^35^. Nonetheless their model utilised YAP-TAZ dependent gene expression events and modulated collagen and myosin levels to control stiffness. Similar to Sun *et al*., this study showed that mechanotransduction and stiffness shape the maintenance of cellular response to mechanical stimulation (even after 4 days). Their model showed that gene-expression network modification allows for prediction of cellular response and tissue level impact. Nonetheless, ECM stiffness does not influence cellular outcome by itself; a degree of system stochasticity is also needed as was alluded to by Peng *et al*. with their stochastic mechanotransduction model^46,51^. This was confirmed by the Mech-ABM which integrates noisy mechanotransduction events even under constant AF_T_ due to activation and deactivation cycles (i.e. feedback loops). A mathematical model by Peng *et al*. demonstrated that stochastic signals influenced YAP-TAZ- mediated gene-expression events and cellular response. It illustrated that particular MSCs’ faith, especially to osteogenic cell lineage, was related to molecular network activation by ECM stiffness value. However, unlike the Mech-ABM, Peng *et al*. model does not integrate mechanotransduction cascades upstream of YAP-TAZ network.

This study demonstrated that mechanosensitive properties, particularly integrins’ mechanosensation, are required for mechanotransduction maintenance. This is a key finding because the major factor inter-linking mechanosensation, mechanotransduction and ECM modulation is yet to be established^52^. MSCs and MLO5 differentiation to OCys, on varied ECM stiffness is a prolonged process^53^, thus mechanosensation and mechanotransduction maintenance is necessary for its completion. The current view is that cytoskeletal mechanosensitivity is related to ECM stiffness^8,46^. However, Mech-ABM illustrated that integrin AF_T_ threshold was of particular importance; a 10-fold increase in integrin mechanosensitivity enhanced mechanotransduction maintenance from 93 ± 10 min to >96 h. This is consistent with the in silico work of Nicolas *et al*. which demonstrated that external force threshold exists for FAs, determining their stability, size, and consequently the cellular response to applied strain^38,54^.

The Mech-ABM also proposed a connection between mechanosensation, mechanotransduction and emergence of mechanical memory. There is accumulating evidence on the role of the TF coactivator YAP/TAZ and microRNAs (miRs) in the emergence of mechanical memory in cells^55–57^. Nonetheless the upstream mediators and the network linking mechanoreceptors and these molecules have not been characterised. The Mech-ABM illustrated that heterogeneity of integrin’s mechanosensitivity contributed to the emergence of mechanical memory. It pointed to pERK as a candidate factor with the establishment of new pERK baseline that was sustainable for longer than 4 days. *In vivo* and *in vitro* observations in neuronal cells support this, where the establishment of new pERK baseline is vital for development of neuronal memory to previous excitations, termed long-term potentiation (LTP). Interestingly osteoblasts and osteocytes possess much of the molecular apparatus required for the same LTP-based memory properties in the CNS^58–60^. Moreover, there are connections between YAP/TAZ and miRs and the MAPK module. YAP/TAZ is recruited by pERK in mechanically induced osteogenic differentiation of MSCs^61^; the expression of the pERK-dependent TF ELK1 is modulated by miR143-3p; while miR21 is involved in ERK pathway negative feedback loops at the level of TFs such as ELK, Spry and MLK1. The latter mediates mechanical memory in lung fibroblasts. In Mech-ABM, alteration of ERK and MEK activation dynamics, via feedback mechanisms, affected the establishment of new pERK baseline, magnitude and oscillatory dynamics. MAPK feedback loops were previously shown to induce “memory modules”^62,63^. Therefore, pERK activation dynamics can be a mechanism that cells utilise to store previous mechanical events and thus oversee the completion of MSC and OB osteogenic differentiation.

## Limitations

This research has generated some plausible predictions on the ways in which mechanical forces influence bone adaptation. We are aware that some assumptions made are different from physiology, and we recognise that they impose limitations on the way that our data can be interpreted. The main simplistic biological assumption is that OBs are the mechanosensing cells. While there is clear evidence for the ability of OBs to respond to loading, it is clear that OCy are likely to be the dominant mechanosensors^64^. Surface strain is a useful predictor of tissue loading, but it is inferior as a sensing system to one in which strain sensors are embedded within the whole of the tissue, and exposed to 3D strain-related information. However, modelling of interactions between OBs and OCys is beyond the scope of the current study.

We also acknowledge that Mech-ABM validation is difficult due to numerous parameters and molecules integrated within the model. However, we have conducted sensitivity analyses on several parameters within the model, which demonstrate its robustness and enhances Mech-ABM credibility, but that work is still ongoing. Furthermore, presently there are aspects within the model which cannot be validated experimentally due to the lack of efficient technology, for instance the precise modulation of a protein activation cycle switch (ACS), and the exact expression of 1% ultrasensitive integrin dimers. Nonetheless, the ability of Mech-ABM to replicate physiological phenomena as emergent behaviour such as the spare receptor concept^43^, the activation dynamics of the Raf-MEK-ERK module as specified by Ferrell *et al*.^42,65^, and the oscillatory behaviour of nuclear-cytoplasmic ERK as illustrated by Shankaran *et al*.^45^, are encouraging.

Other limitations of Mech-ABM include its focus on mechanotransduction propagation through intracellular signalling without integrating interplay with cytoskeletal elements. Therefore, commenting on the role of cytoskeletal elements in the emergence of mechanical memory is also outside the scope of the paper. The OB in the model was an idealised spherical cell, and the ABM rules did not include changes in its morphology as ECM elasticity increased. Though we report the long-term effect of mechanical reciprocity on mechanotransduction dynamics, the ECMp agents increased ECM elasticity in equal manner. However, physiologically ECMps possess different biochemical properties, thus alter ECM elasticity differently.

## Conclusion

The study demonstrated that heterogeneity in the mechanical properties of integrins is fundamental in dictating osteoblast activity due to mechanical stimulation, as well as contributing to the emergence of mechanical memory. The results also forecast that small number of ultrasensitive integrins is capable of sustaining mechanotransduction for prolonged periods, thus this investigation suggests an explanation to why we are far from finding unified mechanical routines to mediate particular cell responses or faiths. These findings open the door to refinement of current mechanoregulation experiments, whereby integration of assays measuring the level of the different integrin dimers, expressed in the plasma membrane, will become essential. Consequently a path towards unified mechanical routines to mediate particular cell responses will be achieved. The work also has the potential to significantly impact scaffold biomaterial designs to create artificial niches, thus influencing the field organs-on-chips.

## Methods

This section is divided into two parts to describe the hybrid model, which is subsequently referred to as the mechano-agent based model (Mech-ABM). The first part describes the ABM that simulates cellular and molecular scales (Fig. 1(a)); while the second describes the mechanical model simulating events at the tissue level (Fig. 1(b)). Communication between the two models is maintained in discrete time-steps. The mechanical model simulates tissue biomechanics and computes numerical force values at every integrin location. The ABM simulates mechanotransduction events within a 3D OB setting in a 3D ECM (osteoid), see Fig. 1.

The Mech-ABM examined the effects of modulating integrins mechanical properties on mechanotransduction and mechanoreciprocity. Specifically it examined the impact of integrins’ mechanosensitivity and its heterogeneity within the integrin population on (1) mechanotransduction dynamics, (2) modulation of tissue material properties and (3) OB response to mechanical stimulation^39^. Mechanosensitivity threshold (MT) was defined as a numerical value of mechanical load that an integrin is exposed to. If AF_T_’s value was equal to or above the MT threshold, it led to integrins activation (Fig. 2).

### The agent-based model (ABM)

A detailed summary of the ABM is provided here. Nonetheless, an extensive description of the ABM is included within the supplementary material and can also be accessed via (web-link). The latter was tailored to the standard protocol describing ABMs: the Overview, Design concepts and Description (ODD)^66^.

The ABM was based on molecular interactions between intracellular molecules (Table 1). Briefly, mechanotransduction is downstream of integrins and triggered by mechanical stimulation imposed at the extracellular matrix (ECM) (Fig. 1(a)). The ERK cascade (Raf-MEK-ERK) is the principal mechanotransduction pathway which links cytoplasmic events to the nucleus and osteogenic genes expression via the interaction of RUNX2 TF. Consequently genes for osteogenic markers such as alkaline phosphatase (ALP), osteopontin (OPN), osteocalcin (OCN) and Bone sialoprotein (BSP) are transcribed and, thereafter, translated into their corresponding proteins (ECMp). ECMps were subsequently deposited in the surrounding ECM adjusting its stiffness. All those molecules were modelled as agents and each deposited ECMp individually increased ECM elasticity (ECMelasticmod) by 0.001. The agents were assumed to be within a well-mixed volume and homogeneously distributed within the cell membrane, cytoplasm and the nucleus. The number of molecules was determined from the literature (supplementary information (1) Initial parameters). The ABM was run for the equivalent to 4 days in real time.

**Table 1:** Number of protein agents included in the ABM at time 0(t_0_).

Molecular Agent	Numbers of agents at t_0_
Integrin	500
FAK	1000
Ras	1000
Raf	32
MEK	3400
Erk	2300
Runx2	24
mRNA - OCN	2
mRNA - OPN	2
mRNA - ALP	2
mRNA - BSP	2
OCN - Protein	1
OPN - Protein	1
ALP - Protein	1
BSP - Protein	1
complex	24
ribosome	600
**Total number of agents**	**8892**

The numbers of proteins were derived from the concentrations of the corresponding proteins which were obtained from the literature (see Supplementary information (1) Initial parameters) The concentrations were converted into moles and then to total number of protein molecules using Avogadro’s number following the procedure outlined in Shuaib *et al*.^69^. The number of agents in active state and distribution in both the cytoplasm and nucleus are shown. The number of mRNA-agents and their corresponding ECM proteins are low at t_0_, however, with time their number significantly increase due to increased production as mechanotransduction propagates.

The ABM was implemented using the generic ABM framework Flexible Large Agent-Based Modelling Environment (FLAME). It schedules agents interactions in discrete time-steps as described previously^67^. A time-step was defined as one second. After the time-step execution, FLAME communicates with the mechanical model. Every molecule-agent is an autonomous communicating X-machine^67,68^ governed by transition functions which are executed serially by the agents.

Transition functions determine molecular behaviours and interactions, controlling molecules’ state transitions from dormant to active states and *vice versa* as modelled previously^69–71^. Transition functions included binding interaction (Fig. S6), movement (Fig. S7) and agents’ re-activation cycles (ACS, Fig. S8). Figure S9 is a stategraph representing the scheduling process between the different transition functions. The model algorithms, detailed state-transition graphs and flowcharts are accessible via UniDrive Link and GitHub.

The agents were heterogeneous and their heterogeneity arises from: agents’ occupancy of different states; spatial separation and compartmentalisation into either the cytoplasm or the nucleus; and stochastic updates of every agent’s global and local variable over time; specifically the Activation Cycle Switch (ACS) variable^67,69,72^. This stochasticity was implemented by random selection of numerical values from either uniform or Gaussian distributions. The distribution selected depended on the modelled parameter, and the agent (Supplementary information (1)). For instance, numerical values of parameters ACS and agent’s interaction radius were extracted from uniform distributions, while rate of protein syntheses was extracted from a Gaussian distribution.

Agents’ common state and global variables are listed in Table 2. Integrins mechanosensitivity threshold is a pivotal state variable and it was examined using four models (Table S1). In models one and two, the integrin population was homogeneous with respect to the mechanosensitivity threshold; while the third and fourth models were heterogeneous (HM). The first mechanosensitivity threshold was set to 10% of AF_T_ (sensitive model (SM)), while the second threshold was set to 1% of AF_T_ (ultrasensitive model (USM)). In the third model, the integrin population was divided into ultrasensitive and sensitive agents with a ratio of 1:10 respectively (10%-HM), while in the fourth model the heterogeneity was changed to 1:100 ratios (1%-HM).

**Table 2:** Global and state variables used in the ABM.

Variable name	Variable type	Functionality	Value and source
**Name**	State	Identifies the molecular agent (e.g. Raf, Runx2 or OPN)	—
**ID**	State	Identifies the molecular agent sequential order	—
**State**	State	Activation state	Adapted from the literature and agent dependent*
**Cartesian coordinates**	State	Expresses the agents coordinates in Cartesian system	Assigned randomly at t_0_ to comply with the homogeneous distribution of molecules within a well-mixed cell^72,76^
**Radian coordinates**	State	Expresses the agents coordinates in radian system	
ACS*	State	Timer to account for feedback loops and thus control dormancy phase	Adapted from the literature and agent dependent*
**interaction radius (iradius)**	State	The radius to allow for interactions between two agents	Adapted from the literature and agent dependent^70^,*
**Cell radius**	Global	Define the outer cell boundary	10 µm^77,78^
**Nuclear radius**	Global	Defines the nucleus cytosol boundary	4 µm^78^
**Time-step**	Global	Every time-step was calibrated to 1 second to account for molecular events	1 s per iteration^69^

The table lists the common variables shared by all agents, however, majority of agents have customised variables, which were listed and can be found in the UniDrive. *For customised variables for specific agents see UniDrive.

Emergent behaviour related to activation dynamics variables of an agent population were monitored and analysed. These were: maximal magnitude of agents’ active state (*E*_*max*_), time to achieve *E*_*max*_ (*t-E*_*max*_), half *E*_*max*_ (*EC*_50_), time to achieve *EC*_50_ (*t-EC*_50_) and magnitude of deposited ECMp levels individually and collectively. The emergence of molecular memory was also of interest. These variables were sampled at every 100^th^ time-step to minimise the signal noise without compromising on detail of the simulation output.

Molecular events occur within 3D space. The distance between two pixels was calibrated to 1 nm^67,69^. Adopting the OB’s physiological volume would have led to agent numbers to be in magnitude of millions; substantially increasing computational costs and model run time. Hence, to minimise this drawback, total cell volume and cellular and nuclear radii were attuned to 1% of the average OB volume^73^. It was previously shown that such an approach had an insignificant influence on altering interaction dynamics in intracellular ABMs^69,74^. The spatiality was partitioned to extracellular environment (ECM), plasma membrane, cytoplasm, nuclear membrane and the nucleus (Fig. 1(a)).

### Mechanical model

The normal stress at the interface of the cell and the ECM can be determined from the expression^75^

1 $${{\boldsymbol{\sigma }}}_{{\boldsymbol{rr}}}=\frac{5\eta k}{[6(2\eta +k)+\frac{4\eta k(71\eta +16k)}{(152\eta +19\eta k+16k)}]}{\sigma }_{xy,\infty }\,{\sin }^{2}\theta \,\sin \,2\varphi $$

Here, *η* = *µ*_C_ /*µ*_M_ is the ratio of shear moduli of the cell (*µ*_C_) and the ECM (*µ*_M_) and *k* = *αR*/*µ*_M_ is non-dimensional ratio relating the stiffness of the interface to the elasticity of the ECM. Specifically, *α* is the spring constant in both normal and tangential directions and the interface is considered to behave as a linear spring. Finally, *R* is the cell radius, *σ*_*xy*,∞_ is the uniform shear stress applied at an infinitely large distance from the cell and *θ* and *ϕ* are spherical polar angles. It is assumed here that this stress is mediated through integrins, which are of equal size. Each of N equal-sized integrins will cover an area equal to 4π*R*^2^/N and a circle possessing the same area has radius *a* = 2 *R*/N^1/2^. If the number of integrins is large, this circular patch is small, and the variation in the normal stress *σ*_*rr*_ over this area can be neglected. For example, with N = 500 integrins the term sin^2^*θ* sin 2*ϕ* varies, on average, less than 7.5% of its maximum value over the cell’s surface. Thus, the force on the integrin is π*a*^2^*σ*_*rr*_ where *a* is the integrin radius. The total number of ECMp deposited regulates the elastic moduli of the ECM. Integrin 3D coordinates are taken from the ABM and used to determine numerical values of force exerted on individual integrin agents (Fig. 1).

### Statistical analysis

Statistical analysis was performed using GraphPad Prism. Mechanotransduction dynamics parameters such as *E*_*max*_, t-*E*_*max*_ and magnitude of pERK were analysed. Analyses between the four integrin mechanosensitivity models (SM, USM, 10%-HM and 1%-HM) were conducted using one way ANOVA, parametrically when the normality of the datasets was confirmed by D’Agostino-Pearson and Shapiro-Wilk normality tests; otherwise nonparametric one way ANOVA was conducted. Post hoc evaluations were via Tukey’s Honest Significant Difference (HSD) test. Analyses between any two models were conducted using Student’s t-test, when dataset was Gaussian. Otherwise, nonparametric analyses were conducted using unpaired t-test with Welch’s correction Mann-Whitney or Wilcoxon signed-rank test as specified. Every model was simulated with an n = 16.

## Supplementary information

Figure legends for the supplementary figures

## Data Availability

The data used in this study is available from https://figshare.shef.ac.uk/account/home#/projects/67427. Please contact the corresponding author for additional information.

## References

[1] Hillam RA, Skerry TM (1995). Inhibition of bone resorption and stimulation of formation by mechanical loading of the modeling rat ulna *in vivo*. Journal of bone and mineral research: the official journal of the American Society for Bone and Mineral Research.

[2] Bikle DD, Halloran BP (1999). The response of bone to unloading. Journal of bone and mineral metabolism.

[3] Turner CH (2009). Mechanobiology of the skeleton. Science signaling.

[4] Ignatius A (2005). Tissue engineering of bone: effects of mechanical strain on osteoblastic cells in type I collagen matrices. Biomaterials.

[5] You J (2001). Osteopontin gene regulation by oscillatory fluid flow via intracellular calcium mobilization and activation of mitogen-activated protein kinase in MC3T3–E1 osteoblasts. Journal of Biological Chemistry.

[6] Xiao Z (2006). Cilia-like Structures and Polycystin-1 in Osteoblasts/Osteocytes and Associated Abnormalities in Skeletogenesis and Runx2 Expression. Journal of Biological Chemistry.

[7] El-Amin SF (2002). Integrin expression by human osteoblasts cultured on degradable polymeric materials applicable for tissue engineered bone. Journal of orthopaedic research: official publication of the Orthopaedic Research Society.

[8] Burridge K, Wittchen ES (2013). The tension mounts: Stress fibers as force-generating mechanotransducers. The Journal of Cell Biology.

[9] Dufour C, Holy X, Marie PJ (2007). Skeletal unloading induces osteoblast apoptosis and targets α5β1-PI3K-Bcl-2 signaling in rat bone. Experimental Cell Research.

[10] Hamidouche Z (2009). Priming integrin alpha5 promotes human mesenchymal stromal cell osteoblast differentiation and osteogenesis. Proc Natl Acad Sci USA.

[11] Fromigue O (2012). Peptide-based activation of alpha5 integrin for promoting osteogenesis. Journal of cellular biochemistry.

[12] Yao W (2013). Reversing bone loss by directing mesenchymal stem cells to bone. Stem cells (Dayton, Ohio).

[13] Shekaran A (2014). The effect of conditional inactivation of beta 1 integrins using twist 2 Cre, Osterix Cre and osteocalcin Cre lines on skeletal phenotype. Bone.

[14] Schneider GB, Zaharias R, Stanford C (2001). Osteoblast integrin adhesion and signaling regulate mineralization. Journal of dental research.

[15] Matziolis D (2011). Osteogenic predifferentiation of human bone marrow-derived stem cells by short-term mechanical stimulation. The open orthopaedics journal.

[16] Di Benedetto A (2015). Osteogenic differentiation of mesenchymal stem cells from dental bud: Role of integrins and cadherins. Stem cell research.

[17] Bandyopadhyay A, Raghavan S (2009). Defining the Role of Integrin αvβ6 in Cancer. Current drug targets.

[18] Seong J (2013). Distinct biophysical mechanisms of focal adhesion kinase mechanoactivation by different extracellular matrix proteins. Proceedings of the National Academy of Sciences of the United States of America.

[19] Xu JK (2012). Optimal intensity shock wave promotes the adhesion and migration of rat osteoblasts via integrin beta1-mediated expression of phosphorylated focal adhesion kinase. The Journal of biological chemistry.

[20] Cheng SL, Lai CF, Blystone SD, Avioli LV (2001). Bone mineralization and osteoblast differentiation are negatively modulated by integrin alpha(v)beta3. Journal of bone and mineral research: the official journal of the American Society for Bone and Mineral Research.

[21] Horton ER (2015). Definition of a consensus integrin adhesome and its dynamics during adhesion complex assembly and disassembly. Nature Cell Biology.

[22] Austen K (2015). Extracellular rigidity sensing by talin isoform–specific mechanical linkages. Nature cell biology.

[23] Yu J, Huang J, Jansen JA, Xiong C, Walboomers XF (2017). Mechanochemical mechanism of integrin clustering modulated by nanoscale ligand spacing and rigidity of extracellular substrates. Journal of the Mechanical Behavior of Biomedical Materials.

[24] Becerra-Bayona S, Guiza-Arguello V, Qu X, Munoz-Pinto DJ, Hahn MS (2012). Influence of select extracellular matrix proteins on mesenchymal stem cell osteogenic commitment in three-dimensional contexts. Acta biomaterialia.

[25] Haugh MG, Vaughan TJ, McNamara LM (2015). The role of integrin αVβ3 in osteocyte mechanotransduction. Journal of the Mechanical Behavior of Biomedical Materials.

[26] Arnold M (2004). Activation of Integrin Function by Nanopatterned Adhesive Interfaces. ChemPhysChem.

[27] Discher DE, Janmey P, Wang Y-l (2005). Tissue Cells Feel and Respond to the Stiffness of Their Substrate. Science.

[28] Engler AJ, Sen S, Sweeney HL, Discher DE (2006). Matrix Elasticity Directs Stem Cell Lineage Specification. Cell.

[29] Barreto S, Clausen CH, Perrault CM, Fletcher DA, Lacroix D (2013). A multi-structural single cell model of force-induced interactions of cytoskeletal components. Biomaterials.

[30] Livne, A., Bouchbinder, E. & Geiger, B. Cell reorientation under cyclic stretching. *Nature Communications* **5**, 3938, 10.1038/ncomms4938, https://www.nature.com/articles/ncomms4938#supplementary-information (2014). 10.1038/ncomms4938 PMC4066201 24875391

[31] Luo Tianzhi, Mohan Krithika, Iglesias Pablo A., Robinson Douglas N. (2013). Molecular mechanisms of cellular mechanosensing. Nature Materials.

[32] Luo T (2012). Understanding the Cooperative Interaction between Myosin II and Actin Cross-Linkers Mediated by Actin Filaments during Mechanosensation. Biophysical Journal.

[33] Nekouzadeh A, Pryse KM, Elson EL, Genin GM (2008). Stretch-activated force shedding, force recovery, and cytoskeletal remodeling in contractile fibroblasts. Journal of Biomechanics.

[34] Chen B, Ji B, Gao H (2015). Modeling Active Mechanosensing in Cell-Matrix Interactions. Annual review of biophysics.

[35] Sun M, Spill F, Zaman Muhammad H (2016). A Computational Model of YAP/TAZ Mechanosensing. Biophysical Journal.

[36] Dingal PC, Dave P, Discher Dennis E (2014). Systems Mechanobiology: Tension-Inhibited Protein Turnover Is Sufficient to Physically Control Gene Circuits. Biophysical Journal.

[37] Kong D, Ji B, Dai L (2008). Stability of Adhesion Clusters and Cell Reorientation under Lateral Cyclic Tension. Biophysical Journal.

[38] Kong F (2013). Cyclic mechanical reinforcement of integrin–ligand interactions. Molecular cell.

[39] Papachroni KK, Karatzas DN, Papavassiliou KA, Basdra EK, Papavassiliou AG (2009). Mechanotransduction in osteoblast regulation and bone disease. Trends in Molecular Medicine.

[40] Pavalko FM (1998). Fluid shear-induced mechanical signaling in MC3T3-E1 osteoblasts requires cytoskeleton-integrin interactions. American Journal of Physiology-Cell Physiology.

[41] Elosegui-Artola Alberto, Bazellières Elsa, Allen Michael D., Andreu Ion, Oria Roger, Sunyer Raimon, Gomm Jennifer J., Marshall John F., Jones J. Louise, Trepat Xavier, Roca-Cusachs Pere (2014). Rigidity sensing and adaptation through regulation of integrin types. Nature Materials.

[42] Ferrell JE, Machleder EM (1998). The Biochemical Basis of an All-or-None Cell Fate Switch in Xenopus Oocytes. Science.

[43] Jacqmin P, McFadyen L, Wade JR (2008). A receptor theory-based semimechanistic PD model for the CCR5 noncompetitive antagonist maraviroc. British Journal of Clinical Pharmacology.

[44] Kholodenko BN (2000). Negative feedback and ultrasensitivity can bring about oscillations in the mitogen-activated protein kinase cascades. European journal of biochemistry / FEBS.

[45] Shankaran H (2009). Rapid and sustained nuclear-cytoplasmic ERK oscillations induced by epidermal growth factor. Molecular systems biology.

[46] Peng X, Huang J, Xiong C, Fang J (2012). Cell adhesion nucleation regulated by substrate stiffness: A Monte Carlo study. Journal of Biomechanics.

[47] Cheng B (2016). An integrated stochastic model of matrix-stiffness-dependent filopodial dynamics. Biophysical journal.

[48] Seong J (2013). Distinct biophysical mechanisms of focal adhesion kinase mechanoactivation by different extracellular matrix proteins. Proceedings of the National Academy of Sciences.

[49] Deeds EJ, Krivine J, Feret J, Danos V, Fontana W (2012). Combinatorial complexity and compositional drift in protein interaction networks. PLoS One.

[50] Mayer BJ, Blinov ML, Loew LM (2009). Molecular machines or pleiomorphic ensembles: signaling complexes revisited. Journal of biology.

[51] Deshpande VS, McMeeking RM, Evans AG (2006). A bio-chemo-mechanical model for cell contractility. Proceedings of the National Academy of Sciences of the United States of America.

[52] Li Z, Lee H, Zhu C (2016). Molecular mechanisms of mechanotransduction in integrin-mediated cell-matrix adhesion. Experimental Cell Research.

[53] Mullen CA, Haugh MG, Schaffler MB, Majeska RJ, McNamara LM (2013). Osteocyte differentiation is regulated by extracellular matrix stiffness and intercellular separation. Journal of the mechanical behavior of biomedical materials.

[54] Nicolas A, Geiger B, Safran SA (2004). Cell mechanosensitivity controls the anisotropy of focal adhesions. Proceedings of the National Academy of Sciences of the United States of America.

[55] Yang Chun, Tibbitt Mark W., Basta Lena, Anseth Kristi S. (2014). Mechanical memory and dosing influence stem cell fate. Nature Materials.

[56] Li Chen Xi, Talele Nilesh P., Boo Stellar, Koehler Anne, Knee-Walden Ericka, Balestrini Jenna L., Speight Pam, Kapus Andras, Hinz Boris (2016). MicroRNA-21 preserves the fibrotic mechanical memory of mesenchymal stem cells. Nature Materials.

[57] Frith JE (2018). Mechanically-sensitive miRNAs bias human mesenchymal stem cell fate via mTOR signalling. Nature Communications.

[58] Skerry TM (2008). The role of glutamate in the regulation of bone mass and architecture. J Musculoskelet Neuronal Interact.

[59] Skerry TM, Taylor AF (2001). Glutamate signalling in bone. Current pharmaceutical design.

[60] Spencer GJ, Genever PG (2003). Long-term potentiation in bone – a role for glutamate in strain-induced cellular memory?. BMC Cell Biology.

[61] Hwang J-H (2017). Nanotopological plate stimulates osteogenic differentiation through TAZ activation. Scientific Reports.

[62] Doncic A (2015). Compartmentalization of a Bistable Switch Enables Memory to Cross a Feedback-Driven Transition. Cell.

[63] Xiong W, Ferrell JE (2003). A positive-feedback-based bistable ‘memory module’ that governs a cell fate decision. Nature.

[64] Skerry TM, Bitensky L, Chayen J, Lanyon LE (1989). Early strain-related changes in enzyme activity in osteocytes following bone loading *in vivo*. Journal of bone and mineral research: the official journal of the American Society for Bone and Mineral Research.

[65] Huang CY, Ferrell JE (1996). Ultrasensitivity in the mitogen-activated protein kinase cascade. Proceedings of the National Academy of Sciences.

[66] Grimm V (2010). The ODD protocol: A review and first update. Ecol. Model..

[67] Coakley S, Smallwood R, Holcombe M (2006). Using x-machines as a formal basis for describing agents in agent-based modelling. Simulation Series.

[68] Kefalas, P., Holcombe, M., Eleftherakis, G. & Gheorghe, M. In *Intelligent agent software engineering* 68–98 (IGI Global, 2003).

[69] Shuaib A, Hartwell A, Kiss-Toth E, Holcombe M (2016). Multi-Compartmentalisation in the MAPK Signalling Pathway Contributes to the Emergence of Oscillatory Behaviour and to Ultrasensitivity. PLOS ONE.

[70] Hao Bai *et al*. Agent-Based Modeling of Oxygen-responsive Transcription Factors in Escherichia coli. *PLOS Comput Biol*, In press (2014). 10.1371/journal.pcbi.1003595 PMC3998891 24763195

[71] Pogson M, Smallwood R, Qwarnstrom E, Holcombe M (2006). Formal agent-based modelling of intracellular chemical interactions. Biosystems.

[72] Pogson M, Holcombe M, Smallwood R, Qwarnstrom E (2008). Introducing Spatial Information into Predictive NF-κB Modelling – An Agent-Based Approach. PLoS ONE.

[73] Beck M (2011). The quantitative proteome of a human cell line. Molecular systems biology.

[74] Rhodes DM, Holcombe M, Qwarnstrom EE (2016). Reducing complexity in an agent based reaction model—Benefits and limitations of simplifications in relation to run time and system level output. Biosystems.

[75] Duan HL, Wang J, Huang ZP, Luo ZY (2005). Stress concentration tensors of inhomogeneities with interface effects. Mechanics of Materials.

[76] Huang CY, Ferrell JE (1996). Ultrasensitivity in the mitogen-activated protein kinase cascade. Proceedings of the National Academy of Sciences of the United States of America.

[77] Yeung CH, Anapolski M, Cooper TG (2002). Measurement of volume changes in mouse spermatozoa using an electronic sizing analyzer and a flow cytometer: validation and application to an infertile mouse model. Journal of andrology.

[78] Yeung CH, Anapolski M, Depenbusch M, Zitzmann M, Cooper TG (2003). Human sperm volume regulation. Response to physiological changes in osmolality, channel blockers and potential sperm osmolytes. Human Reproduction.

