# Supplementary material for: Heterogeneity in The Mechanical Properties of Integrins Determines Mechanotransduction Dynamics in Bone Osteoblasts
Source: Sci Rep. 2019 Sep 11;9:13113. doi: 10.1038/s41598-019-47958-z (PMC6739315; doi:10.1038/s41598-019-47958-z)
Supplement: Supplementary file 1 — Figure legends for the supplementary figures [file 41598_2019_47958_MOESM1_ESM.pdf]

Heterogeneity in The Mechanical Properties of Integrins Determines Mechanotransduction Dynamics in Bone Osteoblasts.

Aban Shuaib[1,2,*], Daniyal Motan[3], Pinaki Bhattacharya[2,3], Alex McNabb[3], Timothy M Skerry[1], Damien Lacroix[2,3]

[1] Department of Oncology and Metabolism, University of Sheffield, Sheffield, UK

[2] Insigneo Institute of *in silico* Medicine, University of Sheffield, Sheffield, UK

[3] Department of Mechanical Engineering, University of Sheffield, Sheffield, UK

***To whom correspondence should be addressed: Aban Shuaib, Department of Oncology and Metabolism, INSIGNEO Institute for in silico Medicine, University of Sheffield, The Pam Liversidge Building, Sheffield, S1 3JD, United Kingdom, Tel: +44 114 22 26072 , Email: aban.shuaib@sheffield.ac.uk.**

Supplementary Figures and Figure legends

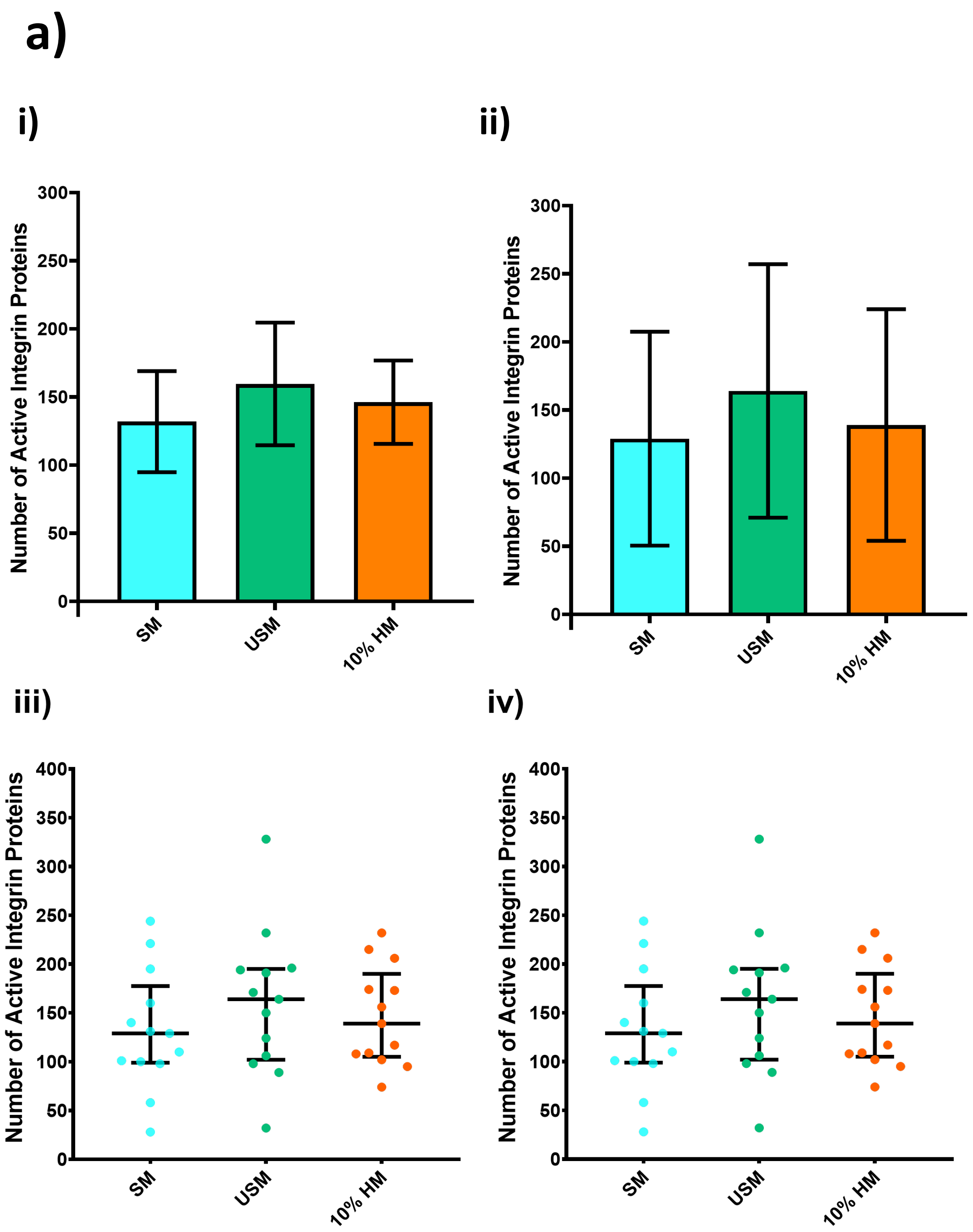

b)

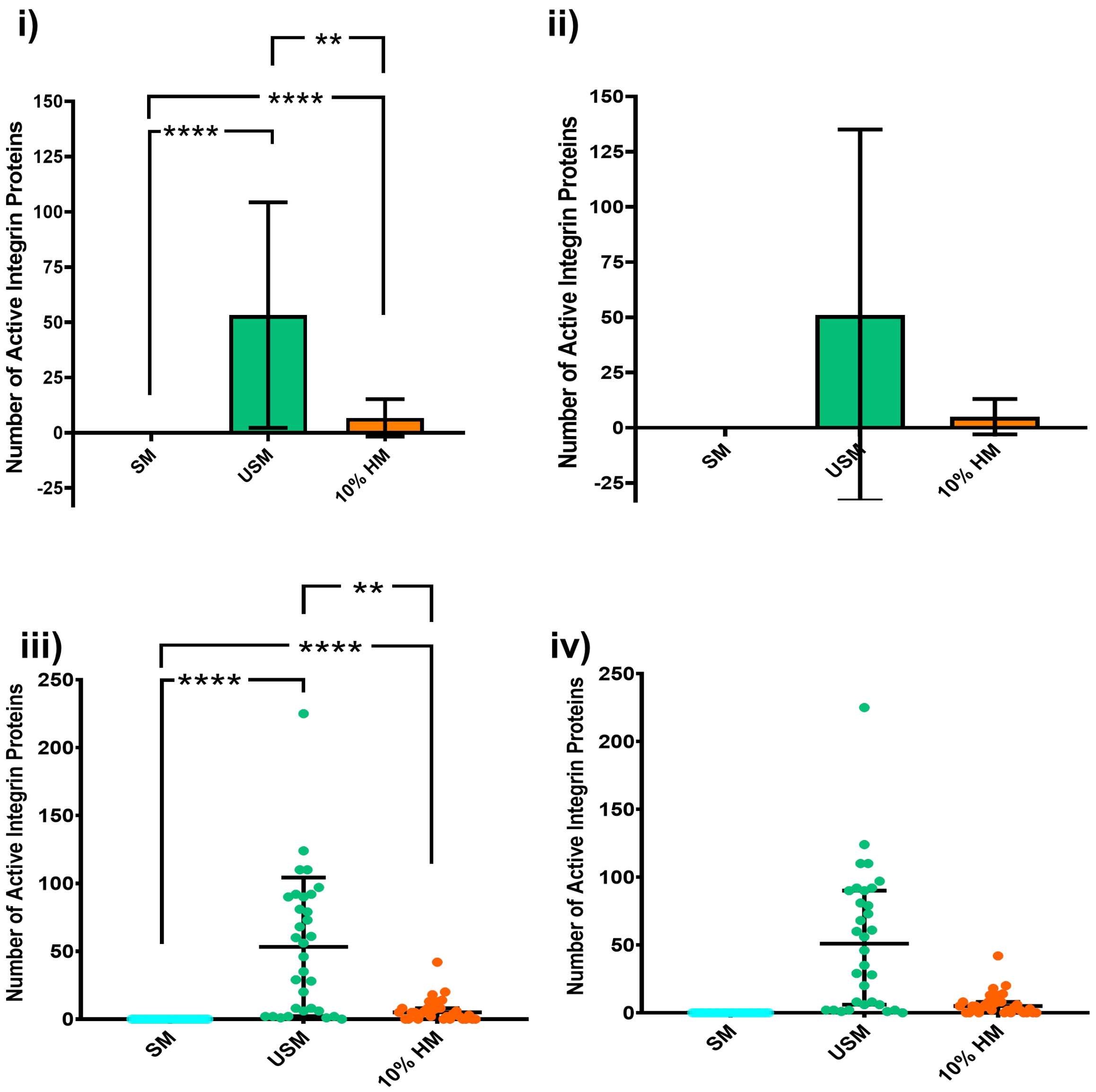
i)
ii)
iii)
iv)
Number of Active Integrin Proteins
SM
USM
10% HM
****
**
150
125
100
75
50
25
0
-25
250
200

Figure S1

Integrin magnitudes comparison during the initial and later activation phases amongst the sensitive, ultrasensitive, and heterogeneous ultrasensitive Mech-ABM. a) The level of active integrins generated at the initial stage mechanical stimulation (0-60 min) illustrating no statistical significance amongst the three models using parametric one-way ANOVA, these were presented with scatter dot plot ((i) & (iii)) representing mean ± SD and median ±IQR respectively. The scatter dot plot show a sample of data from n = 3 simulations. b) Level of active integrins generated at the later stage of mechanical stimulation (400-1440 min) where there is a significant statistical difference between the three models analysed via nonparametric ANOVA (Kruskal-Wallis) . (i) and (iii) show bar and scatter dot plots representing the mean ± SD respectively, while (ii) and (iv) illustrate the same data as median ± IQR. N = 16, **** and ** signify significance where $p(x) < 0.0001$ and $p(x) < 0.0017$.

i)

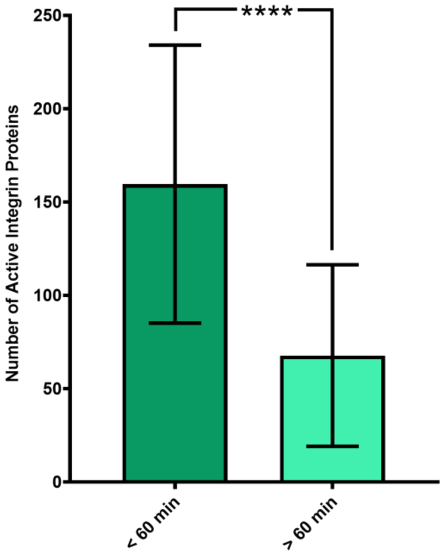
****
250
200
150
100
50
0
Number of Active Integrin Proteins
< 60 min
> 60 min

ii)

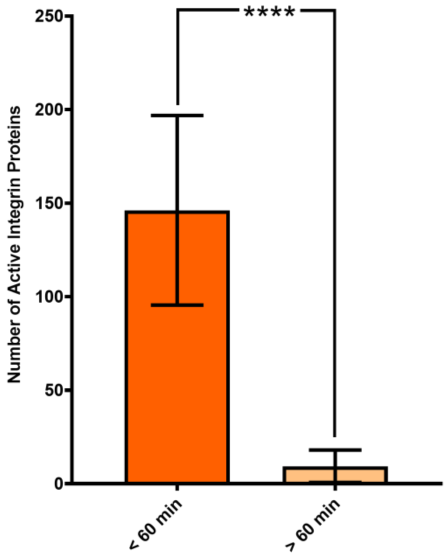
****
250
200
150
100
50
0
Number of Active Integrin Proteins
< 60 min
> 60 min

iii)

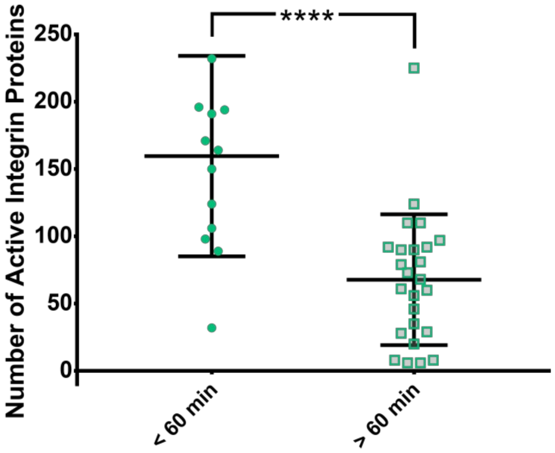
****
250
200
150
100
50
0
Number of Active Integrin Proteins
< 60 min
> 60 min

iv)

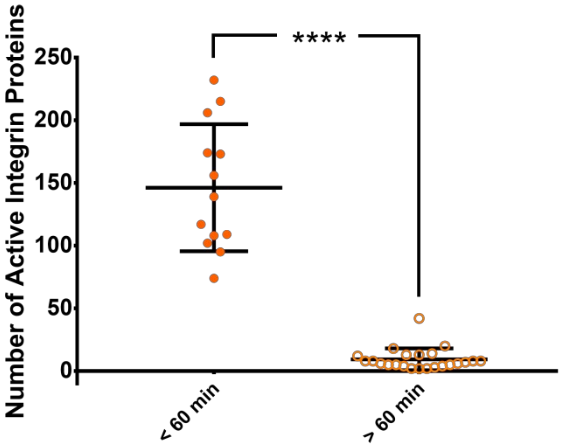
****
250
200
150
100
50
0
Number of Active Integrin Proteins
< 60 min
> 60 min

Figure S2

Magnitude analysis of the level of active integrins during the initial and later activation phases. (i) and (iii) are scatter and dot plots respectively which represent the level of active integrins during the initial activation phase (≤ 60 min) and the consecutive activation phase (> 60 min) in the ultrasensitive model (USM). The data demonstrate that there is a statistical difference between the levels of active integrins during the two phases using both Mann-Whitney test (P value < 0.0001) and Kolmogorov-Smirnov test (P value = 0.0006). The (ii) and (iv) Data from the heterogeneous ultrasensitive model (10% HM) exhibiting the level of active integrins during two activation phase, the initial (≤ 60 min) and the sequential activation phase (> 60 min) showing that the level of integrins were significantly reduced at the later stages of activation using both Mann-Whitney test and Kolmogorov-Smirnov test (both yielded P value < 0.0001). The bar chart reflect the values as mean ± SD while the scatter dot plot exhibits the values as median ± IQR; **** indicates significance where P values were < 0.0001, n = 16 simulations, the scatter dot plots were representations from n = 3 simulations.

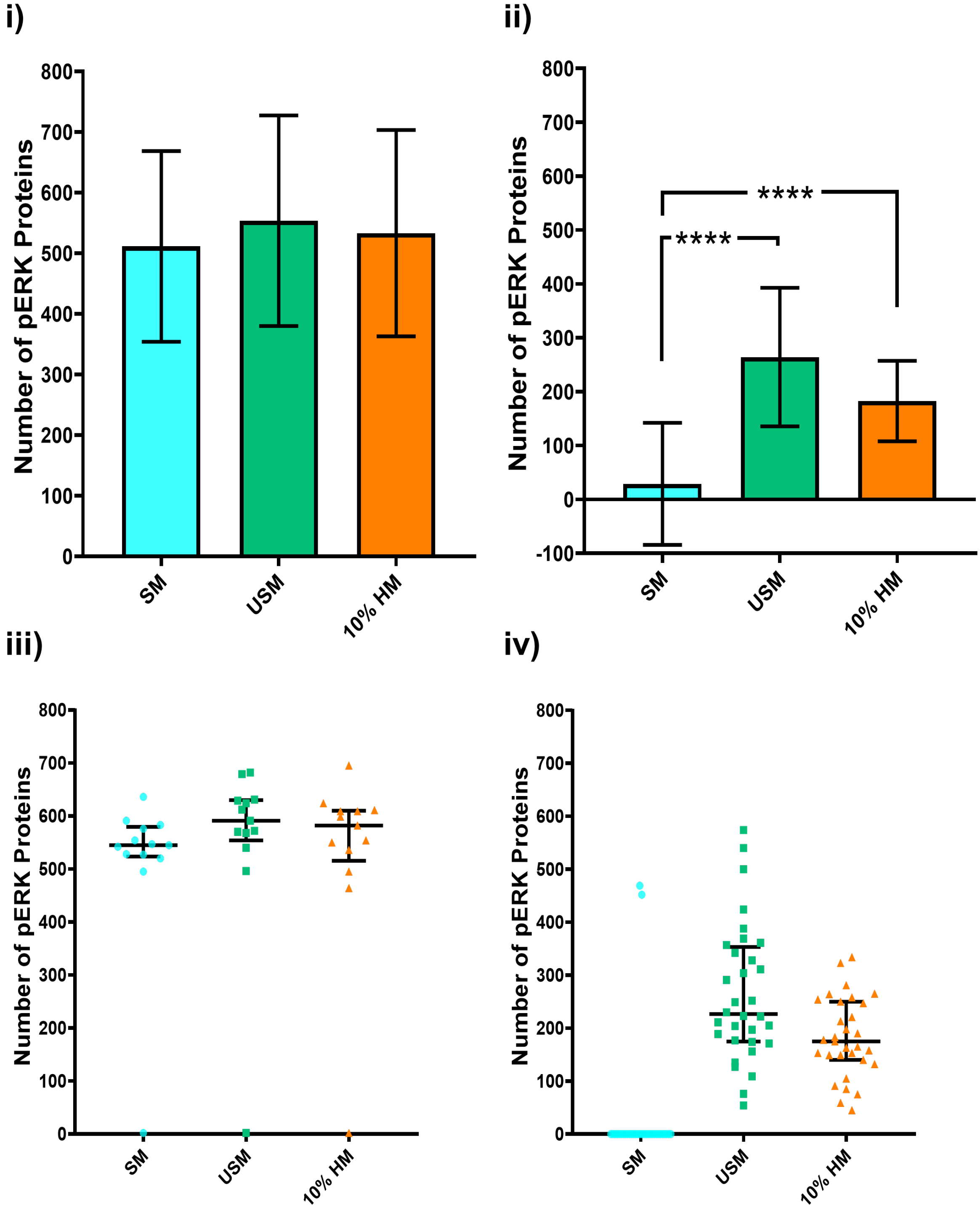
i)
ii)
iii)
iv)
Number of pEPK Proteins
Number of pERK Proteins
800
700
600
500
400
300
200
100
0
-100
****
****
SM
USM
10% HM

Figure S3

Comparison of active (pERK) levels generated by the sensitive, ultrasensitive and heterogeneous ultrasensitive ABMs during the initial activation (0-60 min), and the later activation phases (400-1440 min). (i) and (iii) are histogram and scatter dot representations of pERK levels showing the of generated at the initial stage mechanical stimulation, the scatter dot plot show a sample of data from n = 3 simulations. The data illustrates no statistical significance between the three models using parametric one way. (ii) and (iv) Showing pERK levels at the later activation stages of mechanical stimulation as histogram and scatter dot (400-1440 min). The scatter dot plot show a sample of the full data from n = 3 simulations. In ii) significant statistical differences between the three models were observed using nonparametric one way ANOVA (Kruskal-Wallis test). Both the ultrasensitive and 10% heterogeneous ABMs show significantly higher levels of pERK at the aforementioned activation stage. On the other hand, the difference between the pERK levels produced in the two ultrasensitive ABMs was not as significant. The histograms and scatter dot plots illustrate the same data, nonetheless as mean ± SD and median ± IQR respectively. N = 16, **** implies significance where P value < 0.0001; ** implies a significance where P value = 0.0099.

i)

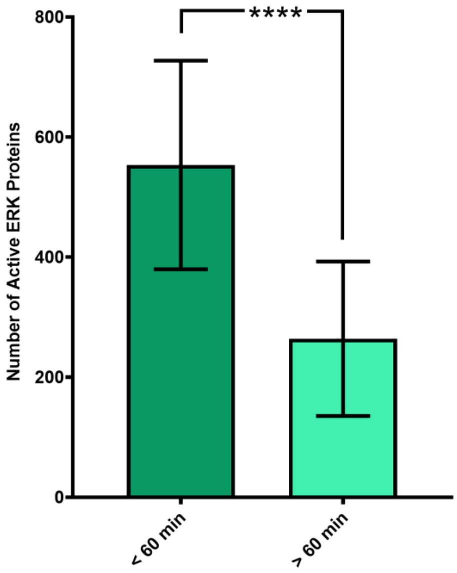
****
Number of Active ERK Proteins
800
600
400
200
0
< 60 min
> 60 min

ii)

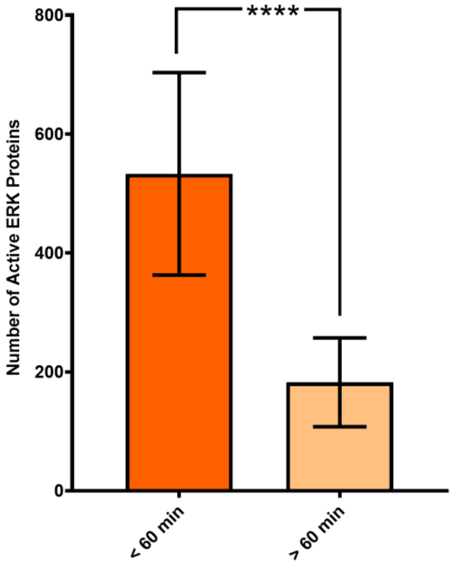
****
Number of Active ERK Proteins
800
600
400
200
0
< 60 min
> 60 min

iii)

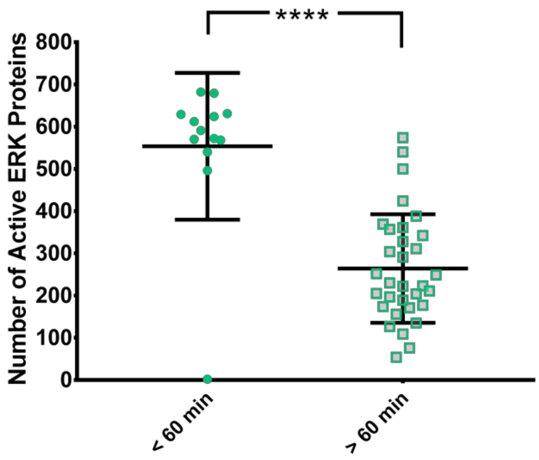
****
Number of Active ERK Proteins
800
700
600
500
400
300
200
100
0
< 60 min
> 60 min

iv)

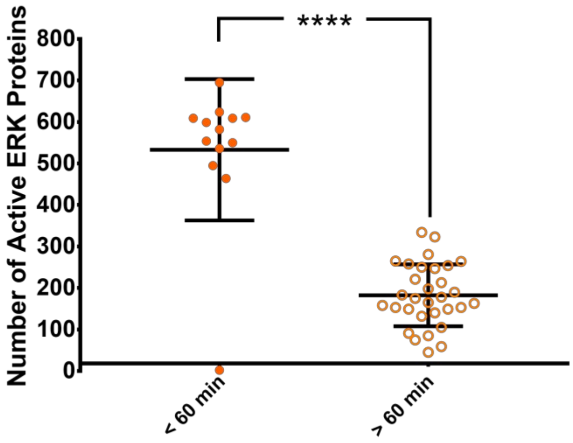
****
Number of Active ERK Proteins
800
700
600
500
400
300
200
100
0
< 60 min
> 60 min

Figure S4

Magnitude analysis of active ERK (pERK) levels during the initial and later activation phases. (i) and (iii) demonstrate the difference in pERK levels during the initial activation phase (≤ 60 min) and the consecutive activation phase (> 60 min) in the ultrasensitive model (USM), where mechanotransduction result in the production of significantly higher levels of pERK in comparison to later stages using both Mann-Whitney test and Kolmogorov-Smirnov test (both yielded P value < 0.0001. (ii) and (iv) Data from the heterogeneous ultrasensitive model exhibiting the level of pERK during two activation phases, the initial (≤ 60 min) and the sequential activation phase (> 60 min) showing that the level of pERK were significantly higher at the early stages of activation using both Mann-Whitney test and Kolmogorov-Smirnov test (both yielded P value < 0.0001. The histograms reflect the values as mean ± SD (i) and ii))while the scatter dot plot exhibits the values as median ± IQR (iii) and iv)); N = 16; **** indicates significance where P value < 0.0001.

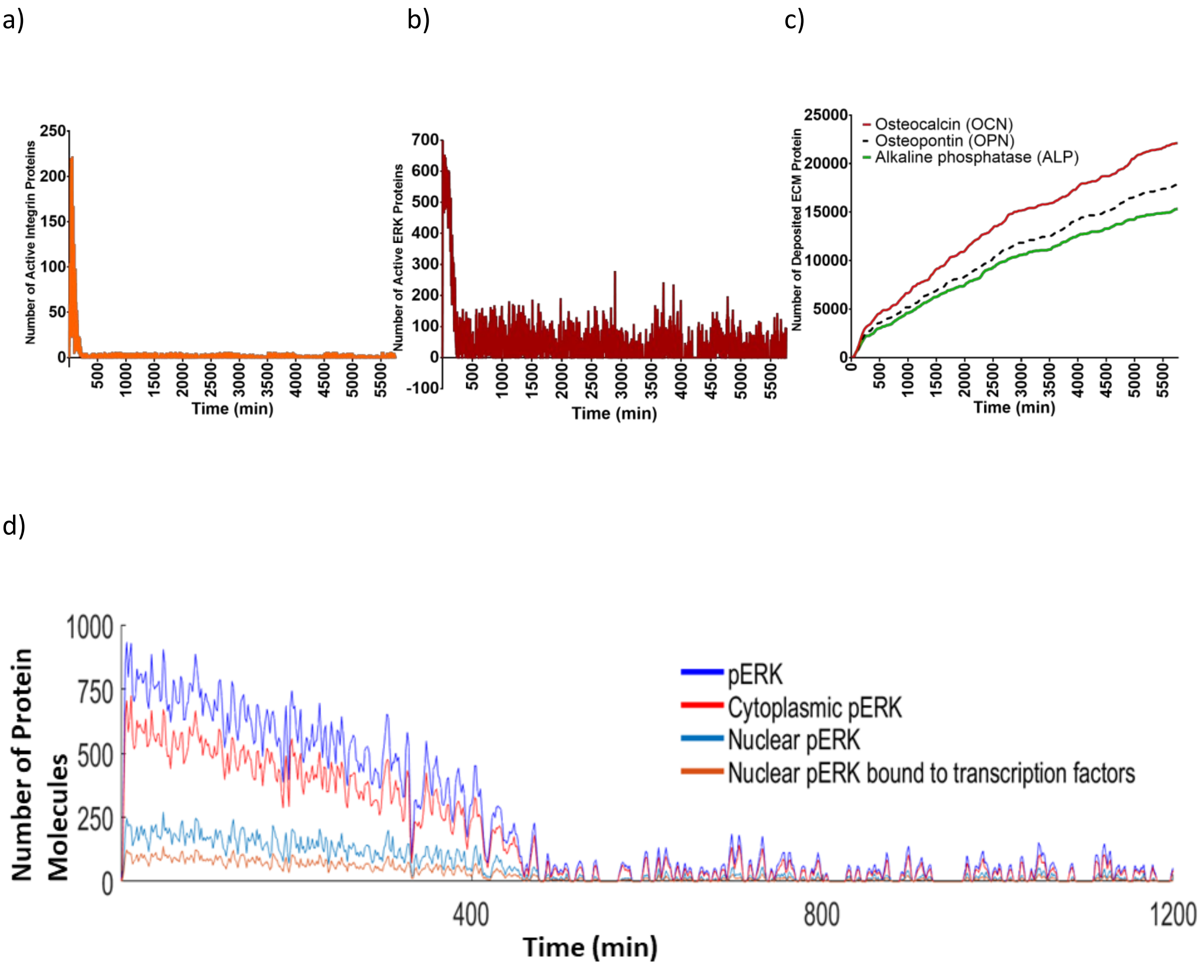

Figure S5

Mechanotransduction dynamics in a 1% heterogeneous ultrasensitive ABM over 4 days. a) Level of active integrins. b) Levels of pERK and formation. c) accumulative ECMp levels. d) Activation behaviour of ERK states in the ABM over 1200 min where the molecular behaviour emerges.

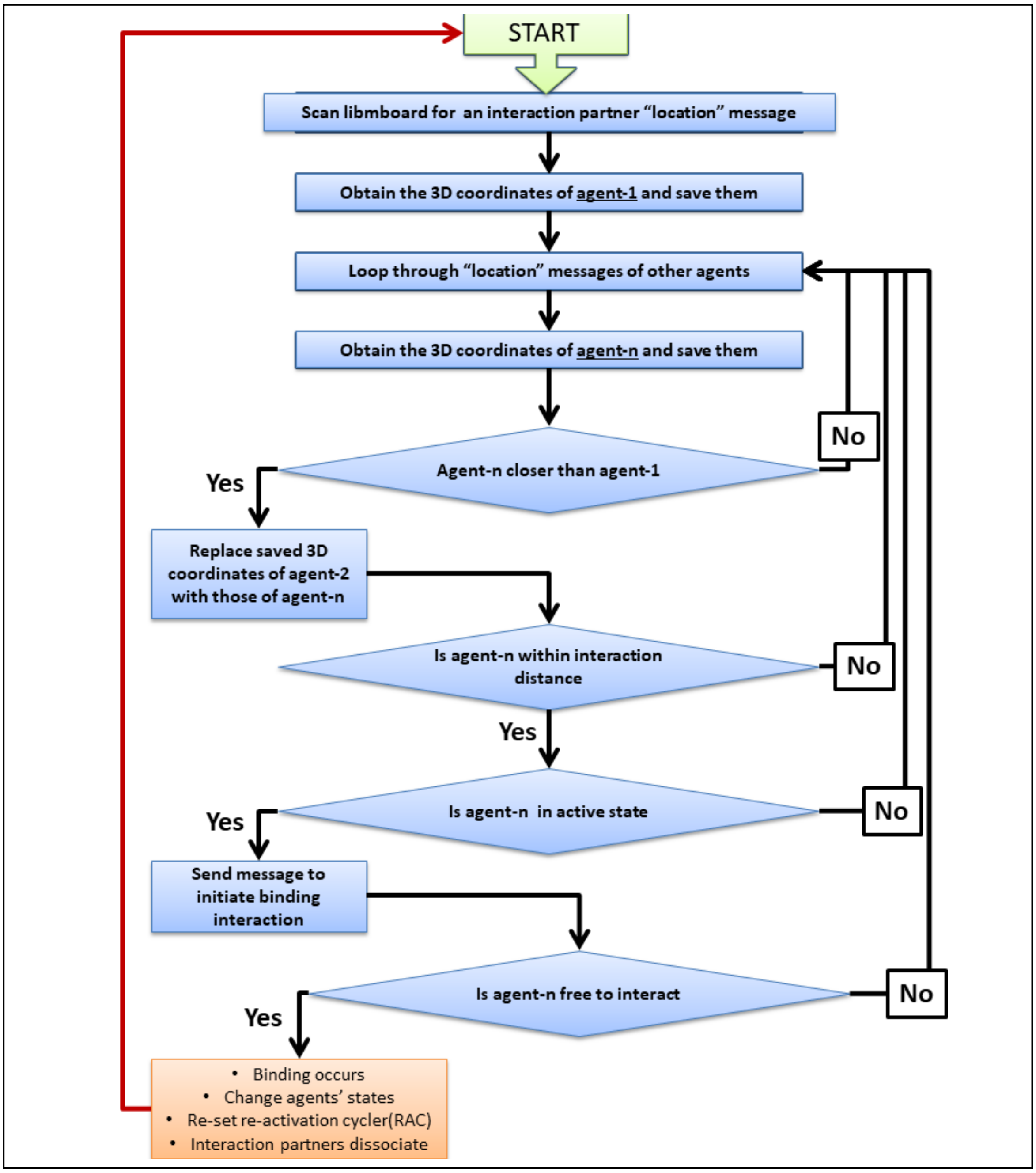
START
Scan libmboard for an interaction partner "location" message
Obtain the 3D coordinates of agent-1 and save them
Loop through "location" messages of other agents
Obtain the 3D coordinates of agent-n and save them
Agent-n closer than agent-1
Yes
No
Replace saved 3D coordinates of agent-2 with those of agent-n
Is agent-n within interaction distance
No
Yes
Is agent-n in active state
Yes
No
Send message to initiate binding interaction
Is agent-n free to interact
Yes
No
Binding occurs
Change agents' states
Re-set re-activation cycler(RAC)
Interaction partners dissociate

Figure S6

Flowchart illustrating the execution of a generic transition function which mediates agents binding interaction. Agent interaction and binding events are fundamental for mediating signal transduction. This was implemented where by agent-agent binding interaction and bond formation are key behaviours. The agent scan its surrounding for the identity of its interaction partners, it screens and loops through all the agents location messages to determine the closest agent. Once determined, the binding interaction ensues. Binding interaction occur if the interacting agents are in the appropriate state and available for binding. Once the conditions are satisfied, state change, re-setting of the ACS and dissociation of complexes (if applicable) are executed and the memory parameters are updated. The time period a protein-agent is in an active state is regulated using the re-activation cycle (ACS, Supplementary materials Box 2), this simulates the intricate balance between positive and negative feedback loops. ACS was simulated stochastically to capture the stochastic nature of the balance between the feedback loops.

Box 2. Agent movement

For every moving agent

Determine new displacement in the polar coordinate:

Determine movement within polar coordinate

Call **current** position with respect to θ (movetheta)

Update movetheta ➔ movetheta + (randomised displacement based on angle range π/10)

Call **current** position with respect to φ (movephi)

Update movephi ➔ movephi + (randomised displacement based on angle range π/10)

Call **current** radian value (mover)

Update mover ➔ average speed + (randomised speed)

Calculate corresponding movement in Cartesian coordinates

Update Cartesian positions with respect to time

Calculate polar position from Cartesian position

If position is beyond the cell membrane

Mirror position back into cell

Update new position

Mirror direction of movement

Update new movement

If position is beyond the nuclear membrane

Mirror position back into the cytoplasm

Update new position

Mirror direction of movement

Update new movement

Figure S7

Pseudo-code illustrating the execution of a generic transition function executing agents Brownian motion movement

<u>Box 3. Agent activation cycle</u>

For every agent after deactivation

If agent is in an inactive state

Check activation cycle switch (ACS) value

If ACS value is ≥ 0

Increment by 1 (ACS -1)

If ACS = 0

Change agent state to active

Reset ACS to a new value (the value is predetermined and is chosen either deterministically or stochastically depending on the agent and the model)

Figure S8

A pseudocode demonstrating agents cycling between active and inactive states. The agent goes through cycles of inactivation and re-activation. This is governed by the Activation Cycle Switch (ACS) memory parameter, which specifies the time an agent remains in a dormant state. Once the agent is in deactivated, the ACS timer commences and increments by one value per iteration of model run. Incrimination of ACS continues until ACS = 0, consequently the agent changes state from inactive to active and the numerical value of ACS is re-assigned. The value equates to the time the agent remains in an inactive state which is determined by the environmental conditions and obtained from the literature. The ACS value is extracted from a uniform distribution

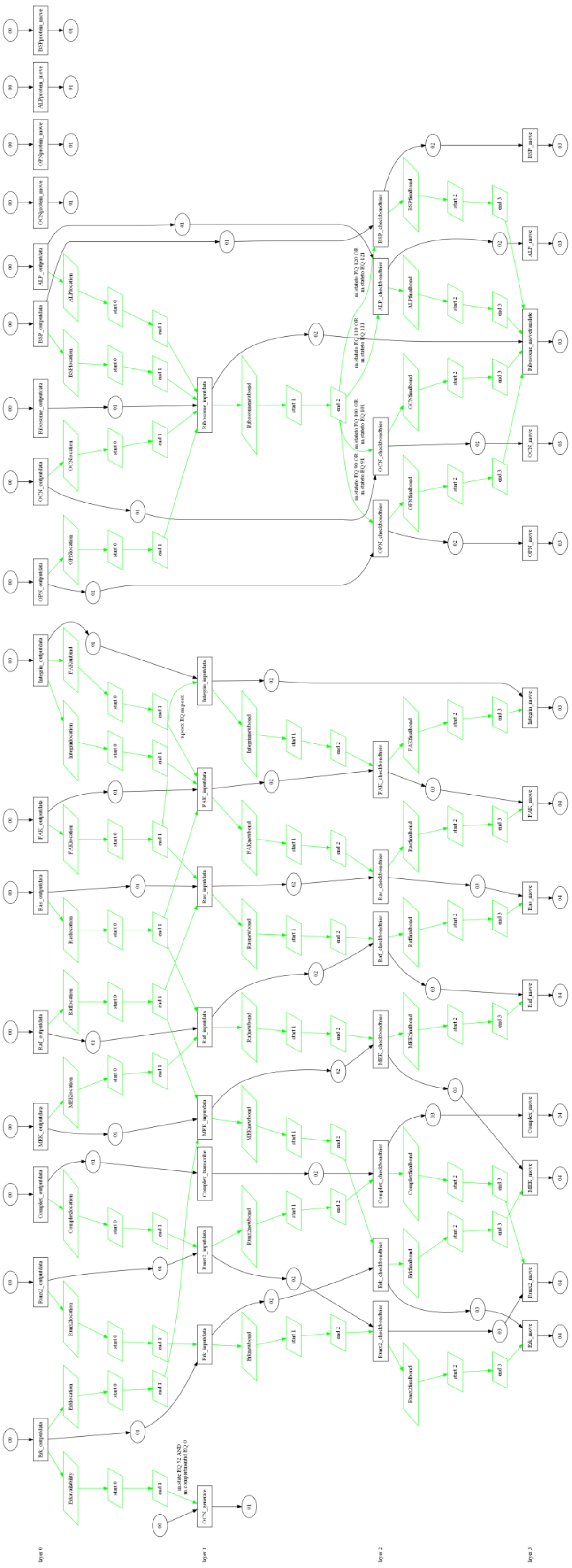

Figure S9

A stategraph representing the communication between the different agent types present in the ABM, the sequence of executing transition functions and which transition functions communicate together via message input and output. Numbered circles donate the execution sequence of an agent's transition functions; the rectangles represent the transition function, green parallelograms represent the messages outputted by a transition function and the arrows point to which transition function the message is used as an input.

Supplementary tables:

Table S1

Mech-ABM name	Abbreviation	Mechanical threshold (as % of applied mechanical load (AFT))	integrin population homogeneity	Heterogeneity ratio (ultrasensitive : sensitive)
**Sensitive**	SM	10%	100%	N/A
**Ultrasensitive**	USM	1%	100%	N/A
**10% Heterogeneous**	10% HM	1 and 10%	N/A	1:10
**1% Heterogeneous**	1% HM	1 and 10%	N/A	1:100

Table S1: Tubular representation of the four Mech-ABMs described in the paper. There are two heterogeneous Mech-ABMs, which differ in their composition ratio of ultrasensitive and sensitive

Table S2

	Rate of ECMp synthesis	
Phase	100% Ultrasensitive Population	10% Ultrasensitive Population
0-60 min	3.1 ± 1	3.3 ± 1.2
1-24 h	8.1 ± 2.1	7 ± 1.2
1-4 days	5.6 ± 0.76	6.6 ± 1.1

Table S2: Rate of extracellular matrix proteins (ECMps) synthesis at different mechanotransduction activation phases in two models of mechanotransduction. The 100% Ultrasensitive Population is the homogeneous USM, where all integrins mechanosensitivity threshold are identical (1% AF_T), while the 10% Ultrasensitive Population was 90% composed of integrin agents with a mechanosensitivity threshold of 10% AF_T and the remaining integrin-agents were with a mechanosensitivity threshold of 1% of integrins 1% AF_T. The rate is shown as mean rate ± SD, n =16.

Supplementary information

Supplementary information (1) Initial parameters

At time 0, the OB that recently migrated into amorphous soft extracellular matrix ECM (i.e. not mineralised and contains no osteogenic ECM proteins) and yet to be exposed to localised mechanical stimulation, thus mechanotransduction was not triggered and all of the intracellular proteins were in dormant state. At this time all agents are homogenously distributed within their corresponding cellular compartments (i.e. within the cytoplasm and the nucleus) and that the agents are well mixed within these compartments (Ferrel et al 1994 and Kholdenko 2000). The numbers of protein agents involved in cascade downstream of integrin receptors were determined from the concentrations specified by Fujioka et al (Kholodenko, 2000, Huang and Ferrell, 1996, Aoki et al., 2008, Ferrell et al., 2009, Levchenko et al., 2000, Li et al., 2014, Widmann et al., 1999) see reference list below. However, this conversion result into a total number of agents in the magnitude of 106s and therefore the simulation become computationally expensive. Consequently, a proportional scaling approach based on cellular volume was conducted based on the work of Shuaib et al[40]. This reduced the magnitude of total agents to 104 and therefore, reduces the simulation expense. The values of ACSs were obtained from the literature, but others were estimated. Mech-ABM was run using two approaches with the initial conditions: the first was using the same initial condition file while with the second some of the initial parameters were changed such as the 3D coordinates of the agents, some of their memory parameters such as ACS value. The stochasticity implemented in Mech-ABM insured that although the same initial conditions were used, however the activation patterns produced from these models were marginally varied.

AOKI, Y., NIIHORI, T., NARUMI, Y., KURE, S. & MATSUBARA, Y. 2008. The RAS/MAPK syndromes: novel roles of the RAS pathway in human genetic disorders. Hum Mutat, 29, 992-1006.

FERRELL, J. E., JR., POMERENING, J. R., KIM, S. Y., TRUNNELL, N. B., XIONG, W., HUANG, C. Y. & MACHLEDER, E. M. 2009. Simple, realistic models of complex biological processes: positive feedback and bistability in a cell fate switch and a cell cycle oscillator. FEBS Lett, 583, 3999-4005.

HUANG, C. Y. & FERRELL, J. E. 1996. Ultrasensitivity in the mitogen-activated protein kinase cascade. Proceedings of the National Academy of Sciences, 93, 10078-10083.

KHOLODENKO, B. N. 2000. Negative feedback and ultrasensitivity can bring about oscillations in the mitogen-activated protein kinase cascades. Eur J Biochem, 267, 1583-8.

LEVCHENKO, A., BRUCK, J. & STERNBERG, P. W. 2000. Scaffold proteins may biphasically affect the levels of mitogen-activated protein kinase signaling and reduce its threshold properties. Proceedings of the National Academy of Sciences, 97, 5818-5823.

LI, J. J., BICKEL, P. J. & BIGGIN, M. D. 2014. System wide analyses have underestimated protein abundances and the importance of transcription in mammals. PeerJ, 2, e270.

WIDMANN, C., GIBSON, S., JARPE, M. B. & JOHNSON, G. L. 1999. Mitogen-activated protein kinase: conservation of a three-kinase module from yeast to human. Physiol Rev, 79, 143-80.

Supplementary information (2) : The agent-based model (ABM)

The ABM description was tailored to the standard protocol describing ABMs: the Overview, Design concepts and Description (ODD) [41]. The ODD describes ABM components, general logic flow and simulation conditions. As ODD was designed for ABM examining social phenomena, a modified version was used herein. These modifications include rearranging ODD by moving Overview to be the second section, yet keeping its subsection "purpose" as the first section describing the ABM.

Purpose

The ABM examined the effects of modulating integrins mechanical properties on mechanotransduction and mechanoreciprocity. Specifically integrins' mechanosensitivity and its heterogeneity within the integrin population on (1) mechanotransduction dynamics, (2) modulation of tissue material properties and (3) osteoblast (OB) response to mechanical stimulation[38]. Mechanosensitivity threshold (MT) was defined as a numerical value of mechanical load an integrin is exposed to. If applied tissue load (AF_T)'s value was equal or above to the MT threshold, it leads to integrins activation (Figure2).

Details

Implementation details

The ABM was implemented using the generic FLAME framework, which was described previously [42]. The model is accessible via **UniDrive Link** and **GitHub**.

Initialisation

The agents were assumed to be within a well-mixed volume and homogeneously distributed within the cell membrane, cytoplasm and the nucleus. Numbers of molecules were determined from the literature (supplementary information (1) Initial parameters). The ABM was run for the equivalent to 4 days in real time. The ABM was run with the following initial conditions.

Molecular Agent	Numbers of agents at t_0
Integrin	500
FAK	1000
Ras	1000
Raf	32
MEK	3400
Erk	2300
Runx2	24
mRNA - OCN	2
mRNA - OPN	2
mRNA - ALP	2
mRNA - BSP	2
OCN - Protein	1
OPN - Protein	1
ALP - Protein	1
BSP - Protein	1
complex	24
ribosome	600
**Total number of agents**	**8892**

Table 1: Number of protein agents included in the ABM at time 0(t_0). The numbers of proteins were derived from the concentrations of the corresponding proteins which were obtained from the literature (see Supplementary information (1) Initial parameters) The concentrations were converted into moles and then to total number of protein molecules using Avogadro's number following the procedure outlined in Shuaib et al 2016[40]. The number of agents in active state and distribution in both the cytoplasm and nucleus are shown. The number of mRNA-agents and their corresponding ECM proteins are low at t_0, however, with time their number significantly increase due to increased production as mechanotransduction propagates. Global and state variables used in the ABM. The table lists the common variables shared by all agents, however, majority of agents have customised variables, which were listed and can be found in the UniDrive

Input Data

The ABM communicates iteration-by-iteration with a mechanical model simulating mechanical events at the tissue level (Figure1). This mechanical model inputted total ECMp number deposited and integrin 3D coordinates. The former was used to update tissue Young's modulus, while the latter was used to determine numerical values of force exerted on individual integrin-agent.

Overview:

Entities, state, variables and scales

These are proteins, nucleic acids (DNA and mRNA) and ribosomes involved in mechanotransduction downstream of the integrins (Table 1). Agents' common state and global variables are listed in Table2. Integrins mechanosensitivity threshold is an important state variable and it was examined using four models (Table 3). In models one and two, the integrin population was homogeneous with respect to the mechanosensitivity threshold; while the third and fourth models were heterogeneous.

Variable name	Variable type	Functionality	Value and source
**Name**	State	Identifies the molecular agent (e.g. Raf, Runx2 or OPN)	-
**ID**	State	Identifies the molecular agent sequential order	-
**State**	State	activation state	Adapted from the literature and agent dependent*
**Cartesian coordinates**	State	expresses the agents coordinates in Cartesian system	Assigned randomly at t_0 to comply with the heterogeneous distribution of molecules within a well-mixed cell [46,47]
**Radian coordinates**	State	expresses the agents coordinates in radian system	
**ACS***	State	Timer to account for feedback loops and thus control dormancy phase	Adapted from the literature and agent dependent*
**interaction radius (iradius)**	State	The radius to allow for interactions between two agents	Adapted from the literature and agent dependent [44]*
**Cell radius**	Global	Define the outer cell boundary	10 μm [48,49]
**Nuclear radius**	Global	Defines the nucleus cytosol boundary	4 μm [49]
**Time-step**	Global	Every time-step was calibrated to 1 second to account for molecular events	1 s per iteration [40]

*** For customised variables for specific agents see UniDrive.**

Table 2: Global and state variables used in the ABM. The table lists the common variables shared by all agents, however, majority of agents have customised variables, which were listed and can be found in the **UniDrive**.

The first model mechanosensitivity threshold was set to 10% of AF_T (sensitive model (SM)); the second the threshold was set to 1% of AF_T (ultrasensitive model (USM)). In the third model, the integrin population was divided into ultrasensitive and sensitive agents with a ratio of 1:10 respectively (10%-HM); in the fourth model the heterogeneity was changed to 1:100 ratios (1%-HM).

The molecular events occur within 3D OB setting within an infinite 3D ECM (osteoid). The distance between two pixels was calibrated to 1 nm. Adapting the OB's physiological volume leads to agents' number in magnitude of millions and thus substantially increasing computational cost and model run time. Consequently, to minimise this drawback, total cell volume and cellular and nuclear radii were attuned to 1% of the average OB volume[50]. This approach was shown previously to be insignificant in altering the interaction dynamics in intracellular ABMs [40,51]. The spatiality was partitioned to extracellular environment (ECM), plasma membrane, cytoplasm, nuclear membrane and the nucleus (Figure1 (a)).

Mech-ABM name	Abbreviation	Mechanical threshold (as % of applied mechanical load (AFT))	integrin population homogeneity	Heterogeneity ratio (ultrasensitive : sensitive)
Sensitive	SM	10%	100%	N/A
Ultrasensitive	USM	1%	100%	N/A
10% Heterogeneous	10% HM	1 and 10%	N/A	1:10
1% Heterogeneous	1% HM	1 and 10%	N/A	1:100

Table 3: Tubular representation of the four Mech-ABMs described in the paper. There are two heterogeneous Mech-ABMs, which differ in their composition ratio of ultrasensitive and sensitive.

Process overview and scheduling

The ABM is run using Flexible Large Agent-Based Modelling Environment (FLAME) that schedules the interaction between the agents in discrete time-steps. Within FLAME, a time-step is defined as one second. Agents are autonomous communicating X-machines where rules (transition functions) are executed serially by the agents depending on state and identity. Figure S1 is a stategraph representing the scheduling process between the different transition functions. For detailed state-transition graphs and flowcharts refer to **UniDrive-Link.** After the time-step execution FLAME communicates with the mechanical model to update the elastic modulus.

Design concepts

Theoretical and empirical background

Emergence: Population activation dynamics of each protein species emerged based on agents binding-unbinding interactions and agent Activation Cycle Switch (ACS) cycles. Binding interactions were based on interaction-partner(s) availability and their state, while activation-inactivation cycles were based on uniform distributions. Figure1 highlight the theoretical bases of the ABM.

Box 1. Agent-agent binding interaction

For every interacting agent

Scan location messages of interaction partners

Obtain the Cartesian coordinates of agent-1 and save

Loop through location messages to obtain the closest interaction partner

If agent-2 is the closest

Replace agent-2 coordinates for agent-1

If agent-2 is within an interaction distance

if interaction partner is in an active state

Send message to initiate binding interaction

If interaction partner is not committed to another binding event

Binding occurs

Box 1: Pseudo-code illustrating the execution of a generic transition function which mediates agents binding interaction. Agent interaction and binding events are fundamental for mediating signal transduction. This was implemented where by agent-agent binding interaction and bond formation are key behaviours. The agent scan its surrounding for the identity of its interaction partners, it screens and loops through all the agents location messages to determine the closest agent. Once determined, the binding interaction ensues. Binding interaction occur if the interacting agents are in the appropriate state and available for binding. Once the conditions are satisfied, state change, re-setting of the ACS and dissociation of complexes (if applicable) are executed and the memory parameters are updated. The time period a protein-agent is in an active state is regulated using the activation cycle switch (ACS, Box 2), this simulates the intricate balance between positive and negative feedback loops. ACS was simulated stochastically to capture the stochastic nature of the balance between the feedback loops.

Individual Decision-Making:

Every agent determined its closest interaction partner (determined by their interaction radius) and its availability for interaction, commenced with binding and state change. Decision making and state transition depends on fulfilment of optimum conditions specified within the transition function (see Box1-3).

Sensing:

All agents, excluding integrins, did not adapt their behaviour to either exogenous or endogenous variables. Integrins receive exogenous stimuli from the mechanical model as a numerical value of the applied force on given spatial location

Agent-agent interaction:

Direct agent-agent binding interactions and mechanisms were predefined in agents transitions functions (Box 1). These relied on Brownian motion in 3D (Box 2) and ACS cycles (Box 3). Brownian motion of membranous protein-agents such as Src, and Ras were restricted to the plasma

Box 2. Agent movement

For every moving agent

Determine new displacement in the polar coordinate:

Determine movement within polar coordinate

Call **current** position with respect to θ (movetheta)

Update movetheta ➔ movetheta + (randomised displacement based on angle range π/10)

Call **current** position with respect to φ (movephi)

Update movephi ➔ movephi + (randomised displacement based on angle range π/10)

Call **current** radian value (mover)

Update mover ➔ average speed + (randomised speed)

Calculate corresponding movement in Cartesian coordinates

Update Cartesian positions with respect to time

Calculate polar position from Cartesian position

If position is beyond the cell membrane

Mirror position back into cell

Update new position

Mirror direction of movement

Update new movement

If position is beyond the nuclear membrane

Mirror position back into the cytoplasm

Update new position

Mirror direction of movement

Update new movement

Box 2: Pseudo-code illustrating the execution of a generic transition function executing agents Brownian motion movement

membrane, while integrin-agents were static. These restrictions were to emulate biological observations [74,75]

Heterogeneity and stochasticity

The agents are heterogeneous and their heterogeneity arises from: agents' occupancy of different states; spatial separation and compartmentalisation into either the cytoplasm or the nucleus; and the stochasticity of ACS [40,42,47]. This stochasticity was implemented by random selection of numerical values from either uniform or Gaussian distributions. The distribution selected depended on the modelled parameter, and the agent. For instance, numerical values of parameters ACS and agent's interaction radius were extracted from uniform distributions. While rate of protein syntheses was extracted from a Gaussian distribution.

Box 3. Agent re-activation cycle

For every agent after deactivation

If agent is in an inactive state

Check ACS value

If activation cycle switch (ACS) value is ≥ 0

Increment by 1 (ACS -1)

If ACS = 0

Change agent state to active

Reset ACS to a new value (the value is predetermined and is chosen either deterministically or stochastically depending on the agent and the model)

Box 3: A pseudocode demonstrating agents cycling between active and inactive states. The agent goes through cycles of inactivation and re-activation. This is governed by the Activation Cycle Switch (ACS) memory parameter, which specifies the time an agent remains in a dormant state. Once the agent is in deactivated, the ACS timer commences and increments by one value per iteration of model run. Incrimination of ACS continues until ACS = 0, consequently the agent changes state from inactive to active and the numerical value of ACS is re-assigned. The value equates to the time the agent remains in an inactive state which is determined by the environmental conditions and obtained from the literature. The ACS value is extracted from a uniform distribution

Observation

For model analysis, activation dynamics variables of an agent population were recorded; these include: maximal magnitude of agents' active state (E_{max}), time to achieve E_{max} ($t\text{-}E_{max}$), half E_{max} (EC_{50}), time to achieve EC_{50} ($t\text{-}EC_{50}$) and magnitude of deposited ECMp levels individually and collectively. These were also the emergent behaviours which were monitored and analysed, in particular the emergence of molecular memory is of interest. We sampled these variables at every 100th time-step to minimise the signal noise without compromising on detail of the simulation output.

Supplementary information (3) FLAME

Briefly, agents were simulated as communicating X-machines relying on memory parameters, transition functions, transition states and communicating messages. Memory parameters were written in XMML (X-machine Markup Language) which hold agents memory variables, agents messages memory parameters and definition of the agents environment. The respective transition functions are coded in a separate function file coded in C. These XMML and transition function files are defined by the user. FLAME utilises these codes to generate an iterative-based executable model. This is achieved by parsing the XMML files into simulation source code codes accessible by the transition functions. The agents communicate via messages, which they send (output message) and access (input message) to a message library (libmboard). Inputting and outputting messages is specified within transition functions. These messages are accessed by agents per iterations. In our model, agents only access the messages produced by their interaction partners. FLAME run in a sequential manner (up to the iteration no. specified by the user) and allow storage of information per iteration in automatically generated XML files, referred to as the ABM's memory files. All models were executed on Iceberg High Performance Computing Cluster based at the University of Sheffield.
